# L-Theanine Alleviates IMQ-Induced Psoriasis Like Skin Inflammation by Downregulating the Production of IL-23 and Chemokines

**DOI:** 10.3389/fphar.2021.719842

**Published:** 2021-07-26

**Authors:** Yaohan Xu, Jiang Zhu, Jingyi Hu, Ziqi Zou, Yueling Zhao, Lihua Lai, Ping Xu, Yinjing Song, Hao Cheng

**Affiliations:** ^1^ Department of Dermatology and Venereology, Sir Run Run Shaw Hospital, Zhejiang University School of Medicine, Hangzhou, China; ^2^ Institute of Immunology, Zhejiang University School of Medicine, Hangzhou, China; ^3^ Tea Research Institute, College of Agriculture and Biotechnology, Zhejiang University, Hangzhou, China

**Keywords:** L-Theanine, psoriasis, IL-23, chemokines, dendritic cells

## Abstract

Psoriasis, the most common skin inflammatory disease, is characterized by massive keratinocyte proliferation and immune cell infiltration into epidermis. L-Theanine (L-THE), a nonproteinogenic amino acid derived from green tea (Camellia sinensis), has been proved to possess the properties of anti-inflammatory, antidepressants and neuroprotective. However, whether L-THE has a therapeutic effect on psoriasis is still unknown. In this study, we found that the epidermal thickness and inflammatory response were significantly reduced in Imiquimod (IMQ)-induced psoriasis mice by applying with L-THE on mice skin. The expression of proliferation and inflammation associated genes such as *keratin 17*, *IL-23* and *CXCL1-3* was also downregulated by L-THE. Furthermore, L-THE inhibited the production of IL-23 in dendritic cells (DCs) after IMQ treatment, and decreased the levels of chemokines in keratinocytes treated with IL-17A by downregulating the expression of IL-17RA. RNA-seq and KEGG analysis revealed that L-THE significantly regulated the expression of IL-17A and NF-κB signaling pathway-associated genes. Metabolomics analysis displayed that L-THE promoted propanoate metabolism which has been reported to inhibit the activity of TH17 cells. Therefore, our results demonstrated that L-THE significantly decreases the levels of IL-23 and chemokines, and attenuates IMQ-induced psoriasis like skin inflammation by inhibiting the activation of NF‐κB and IL-17A signaling pathways, and promoting the propanoate metabolism. Our findings suggest that topical applied L-THE can be used as a topical drug candidate for the treatment of psoriasis or as an adjuvant treatment of ustekinumab or secukinumab to prevent the relapse of psoriasis.

## Introduction

Psoriasis is one of the most common immune-mediated chronic inflammatory skin disorders, affecting about 2–3% of the population worldwide (Gonzalez-Cantero et al., 2021). It is characterized by massive keratinocyte proliferation, brisk immune cell infiltration and is considered a metabolic syndrome (Yamanaka et al., 2021). The etiology of psoriasis involves both the innate and adaptive immune system which result in the hyperproliferation, abnormal differentiation of keratinocytes and neovascularization (Martinez-Moreno et al., 2020). In psoriasis, characteristic skin lesions contain abnormal infiltration of DCs, T cells, neutrophils and macrophages (Niculet et al., 2020; Damiani et al., 2021). However, the specific mechanism of psoriasis development is still unclear and the strategies to cure psoriasis are also being sought.

Recently, the interleukin 23 (IL-23)/T-helper 17 (Th17) immune axis has been identified as a key driver of psoriasis disease pathogenesis (Di Cesare et al., 2009; Egeberg et al., 2020). A large number of studies have shown that the deregulated production of IL-23 secreted by DCs can initiate the progression of psoriasis (Lillis et al., 2010; Kagami, 2011). These discoveries have also promoted the development of biologics for psoriasis, such as Ustekinumab targeting IL-23, Secukinumab targeting IL-17 which have been approved by the US Food and Drug Administration to treat psoriasis, and result in dramatic improvements in approximately 80–90% of psoriasis patients (Campa et al., 2016; Boutet et al., 2018; Ghoreschi et al., 2021). However, the recurrence of psoriasis is also occurred after biologics therapies (No et al., 2017; Geller et al., 2018). Therefore, there is always a lack of effective measures to prevent the recurrence of psoriasis.

L-Theanine (L-THE), a water-soluble non-protein amino acid, is predominantly found in *Camellia sinensis* and recognized as one of the critical metabolites contributing to the quality and health benefits of teas (Sharma et al., 2018). L-THE can cross the blood brain barrier *via* L-amino acid transporter system to reduce stress and inhibit anxiety (Katasonov, 2018; Shojaei-Zarghani et al., 2021). Safety evaluation studies indicate that L-THE is well tolerated at a high dose without any toxic effects (Williams et al., 2020). Currently, L-THE is a known agent for improving sleep disturbances, and it also has effects on reducing stress and anxiety-like symptoms (Williams et al., 2019). In addition, several studies have reported that L-THE has multiple pharmacological and physiological functions, including antioxidant, anti-inflammatory, and immune response regulation (Wang et al., 2018a; Li et al., 2018; Zhang et al., 2019a). It has also been reported that L-THE attenuates the 2-O-tetradecanoylphorbol-13-acetate (TPA)-induced acute skin inflammation (Zeng et al., 2018). However, whether L-THE has an effect on psoriasis and its potential mechanism remain unknown.

In this study, we found that topical applied L-THE significantly alleviated the epidermal thickness and inflammatory response, and inhibited the expression of the proliferation and inflammation associated genes in IMQ-induced psoriasis mice. Furthermore, L-THE inhibited the production of IL-23 and IL-17A in dendritic cells and keratinocytes , respectively. RNA-seq and metabolomics analysis revealed that L-THE significantly regulates the expression of *IL-17A* and *NF-kB* signaling pathway associated genes, while promoting propanoate metabolism. Therefore, our results demonstrate that L-THE significantly downregulates the production of IL-23 and chemokines, and alleviates the IMQ-induced psoriasis like skin inflammation by restraining the activation of NF‐κB and IL-17A signaling pathway, and promotes the propanoate metabolism, suggesting that L-THE can be used to treat psoriasis in clinical.

## Materials and Methods

### Mice and Reagents

C57BL/6 mice were purchased from Shanghai SLAC Laboratory Animal. All mice were housed at the Zhejiang University Laboratory Animal Center under specific pathogen-free conditions. All the animal experiments were performed with relevant guidelines and regulations approved by the Institutional Animal Care and Use Committee at Zhejiang University. This study was approved by the Ethics Committee of Sir Run Run Shaw Hospital of Zhe Jiang University School of Medicine (approval no. 20200717-015).

IMQ (tlrl-imq) was purchased from Invivo Gen. Recombinant mouse GM-CSF (315-03) and IL-17A (200-17) were from PeproTech. TRIzol (15596018) was purchased from Thermo Fisher. ReverTra Ace qPCR RT Kit (FSQ-201) was from Toyobo. SYBR Green master Rox (04707516001) was from Roche. IL-23 ELISA kit (433704) was purchased from Biolegend and CXCL1 kit (ab216951) was from Abcam.

### Animal Models of Psoriasis and L-THE Topical Treatment

For the IMQ-induced mouse model of psoriasis, male C57BL/6 mice (8 weeks-of-age) were subjected to a daily topical dose of 62.5 mg IMQ cream (5%) (Aldara, 3M Pharmaceuticals) on the shaved back skin or ear for five consecutive days. L-THE was obtained from the Department of Tea Science, Zhejiang University and dissolved in the deionized water for 10 mM or 100 mM, then were topical applied with 10 mM or 100 mM L-THE on the skin for twice per day.

### RNA Extraction, Library Construction and Sequencing

Total RNA from mouse tissues were extracted using TRIzol (Takara). Preparation of the library and transcriptomic sequencing were carried out using Novaseq™ 6000 (LC-Bio Technology, Hangzhou, China) following the vendor’s recommended protocol. Mapping of 150-bp paired-end reads to genes was done using HTSeq software (version 0.6.0), and fragments per kilobase of transcript per million fragments mapped (FPKM) were also analyzed. The differentially expressed genes (DEGs) were selected with fold change >2 or fold change <0.5 and p value <0.05 by R package edgeR (https://bioconductor.org/packages/release/bioc/html/edgeR.html) or DESeq2 (http://www.bioconductor.org/packages/release/bioc/html/DESeq2.html). Gene ontology (GO) enrichment analysis was calculated using an equation previously described (Wang et al., 2018b). The Kyoto Encyclopedia of Genes and Genomes (KEGG) pathway enrichment was used t to annotate and visualize the signaling pathways of DEGs. The gene expression and pathway analyses were performed by the LC-Bio company (Hangzhou, China). The RNA-Seq data are available in the NCBI’s SRA (Sequence Read Archive) repository (BioProject ID: PRJNA742565).

### Bone Marrow-Derived Dendritic Cells Isolation and Culture

Bone marrow cells were isolated from mice and cultured in RPMI-1640 medium with 10% FBS and 20 ng/ml GM-CSF. Fresh medium containing 20 ng/ml GM-CSF was added in the cultured cells at day 3, then removed half of the media and added fresh medium containing 20 ng/ml GM-CSF at day 5. BMDC can be harvested on day 6. All cells were cultured at 37°C and 5% CO_2_.

### RT-PCR

Total RNA was extracted from mouse skin tissues, DC or keratinocytes using TRIzol Reagent (Takara) according to the manufacturer’s directions. Subsequently, single-strand cDNA synthesis was performed using a Tyobo reverse transcription kit. qRT-PCR was performed using SYBR Green Master Rox (Roche) on a CFX-96 (Bio-Rad) or 480II (Roche) Real-Time PCR System, Results were normalized to Gapdh and quantification was carried out using the 2^–△△Ct^ method. The primers of candidate genes for qRT-PCR were showed in the Supplementary Table S1.

### Immunohistochemistry Staining

Mouse back skin or ear tissues were fixed with 4% formaldehyde, immunohistochemical staining was performed by the Histomorphology Platform of Zhejiang University, with the standard protocol performed according to the manufacturer’s instructions. Stained sections were captured using an Olympus microscope (IX73).

### Enzyme Linked Immunosorbent Assay

BMDC were treated with 4 μg/ml IMQ for 24 h or mouse primary keratinocytes were stimulated with 100 ng/ml IL-17A for 24 h, and supernatant was taken for IL-23 and IL-17A testing according to the manufacturer’s protocols.

### Metabolite Extraction, LC-MS Analysis, and Data Processing

The back-skin tissues from mice treated with IMQ or L-THE were thawed on ice, and metabolites were extracted from 20 µl of each sample using 120 µl of precooled 50% methanol buffer. Pooled quality control (QC) sample were also prepared by combining 10 μl of each extraction mixture. All samples were detected by a TripleTOF 5600 Plus highresolution tandem mass spectrometer (SCIEX, Warrington, United Kingdom) with both positive and negative ion modes. Chromatographic separation was performed using an ultraperformance liquid chromatography (UPLC) system (SCIEX, United Kingdom). The acquired LC-MS data pretreatment was performed using XCMS software.

### Statistical Analysis

All the statistical analysis was expressed as the mean ± SD and performed using Prism 6 (GraphPad Software). Statistical significance was evaluated by two-way ANOVA. All experiments are repeated at least three times independently. Asterisk coding is indicated in Figure legends as *, *p* < 0.05; **, *p* < 0.01; ***, *p* < 0.001.

## Results

### L-THE Decreases Epidermal Thickness in Mice With IMQ-Induced Psoriasis Like Skin Inflammation

**FIGURE 1:**
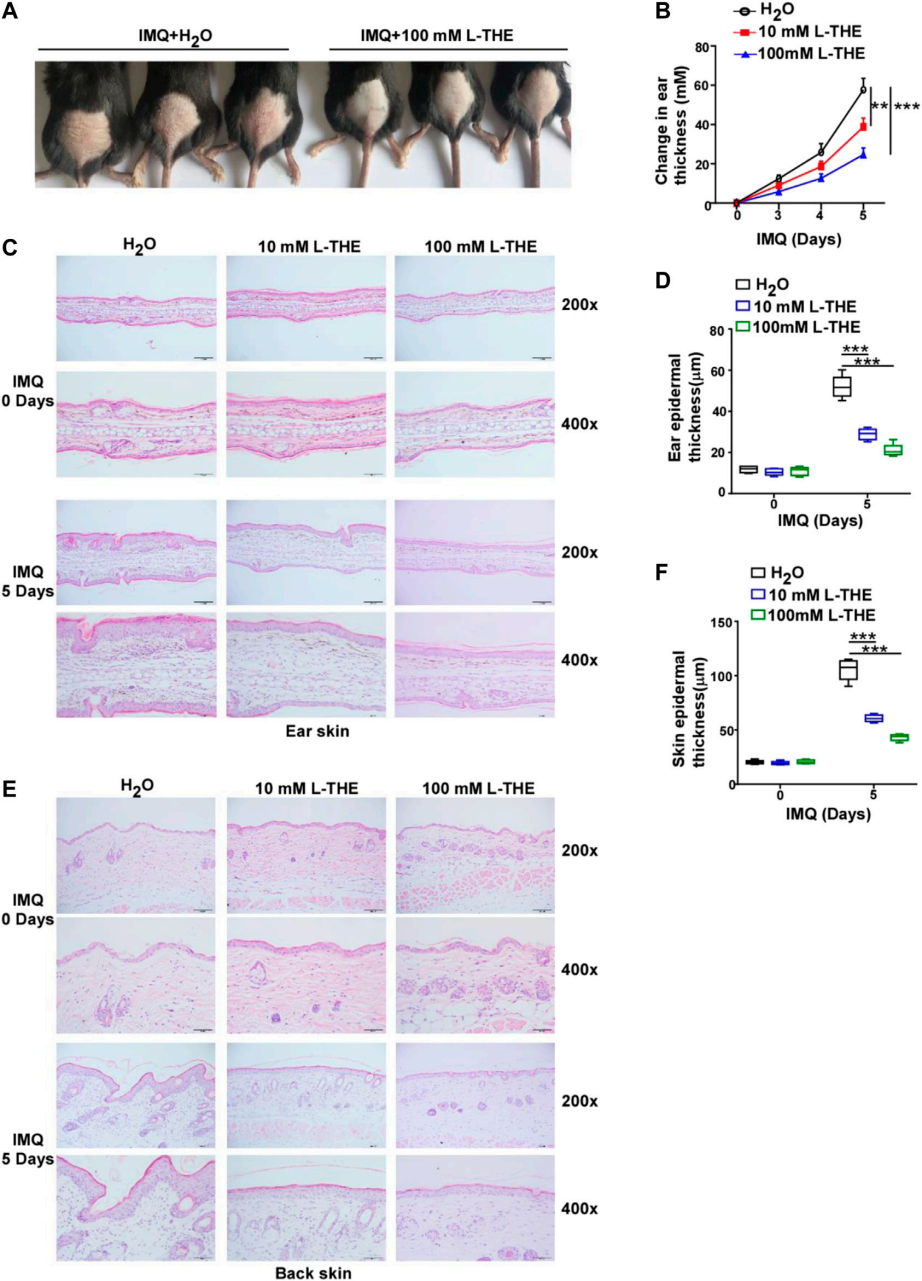
L-THE decreases epidermal thickness in mice with IMQ-induced psoriasis like skin inflammation. **(A)** C57BL/6 mice (*n* = 6 per group) were subjected to a daily topical dose of IMQ cream on the shaved back or ear for five consecutive days, and were topical applied with 10 mM or 100 mM L-THE on the skin for twice per day. **(B)** Mice ear thickness was measured relative to the contralateral ear from IMQ-induced psoriasis mice treated with L-THE or H_2_O. **(C)** H&E staining ear skin sections obtained from IMQ-induced psoriasis mice treated with L-THE or H_2_O for 5 days. **(D)** The ear epidermal thickness was measured in **(C)** by the software ImageJ. **(E)** H&E staining back skin sections obtained from IMQ-induced psoriasis mice treated with L-THE or H_2_O for 5 days. **(F)** The ear epidermal thickness was measured in **(D)** by the software ImageJ. Data is representative of three independent experiments. *p* values are determined by two-way ANOVA. **p < 0.01, ***p < 0.001.

To investigate whether L-THE could regulate the psoriasis-like skin inflammation, L-THE (10 mM or 100 mM) was applied to the back skin or ears of mice were treated with IMQ cream (5%) for 5 days. We found that L-THE significantly attenuated the symptom of psoriasis-like skin inflammation (Figure 1A). The change in thickness of ear was also decreased after treatment with L-THE (Figure 1B). Compared to the control mice, L-THE-treated mice showed less severe swelling, epidermal acanthosis, skin inflammation, and proliferation of keratinocytes in the ear skin subjected with IMQ cream (Figure 1C). The L-THE-treated mice showed a lower level of ear epidermal thickness than control mice after IMQ cream treatment for 5 days (Figure 1D). We also found that the swelling, epidermal acanthosis, skin inflammation, and proliferation of keratinocytes were also extenuated after L-THE treatment in the back skin after treatment with IMQ cream for 5 days (Figure 1E). The epidermal thickness of back skin was significantly reduced after L-THE treatment (Figure 1F). Therefore, our results demonstrated that L-THE attenuates the epidermal thickness in mice with IMQ-induced psoriasis.

### L-THE Regulates the Expression of Genes Related to the Proliferation and Differentiation of Keratinocytes

**FIGURE 2:**
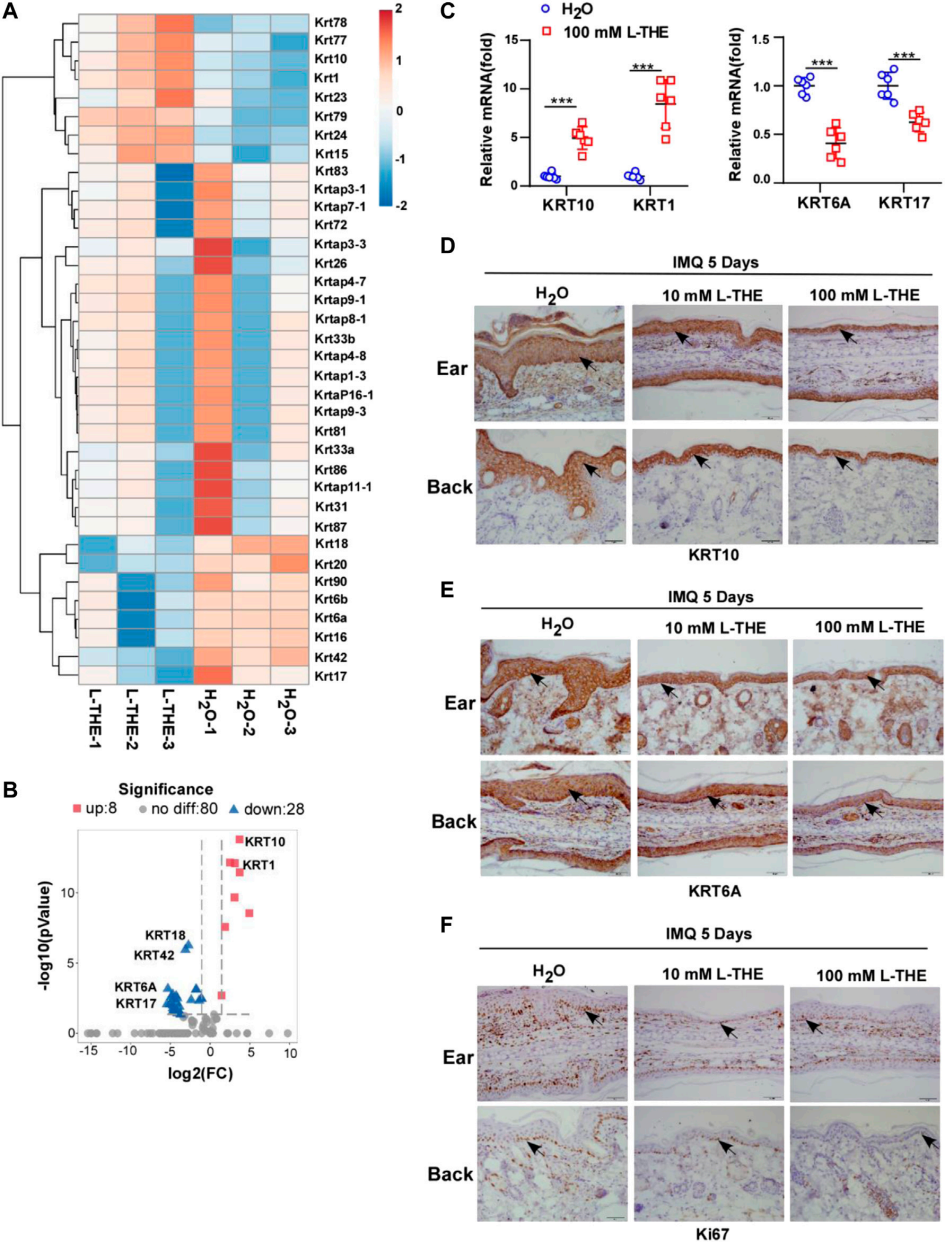
L-THE regulates the expression of genes related to the proliferation and differentiation of keratinocytes. **(A,B)** Heatmap **(A)** and Volcano plot. **(B)** showed the expression of KRT genes based on RNA-seq data from IMQ-induced psoriasis mice treated with 100 mM L-THE or H_2_O for 5 days. **(C)** RT-PCR analysis of the mRNA levels of KRT10, KRT1, KRT6A, and KRT17 in IMQ-induced psoriasis mice (*n* = 6 per group) treated with 100 mM L-THE or H_2_O for 5 days. **(D,F)** IHC staining of KRT10, KRT6A and Ki67 in ear or back skin sections obtained from IMQ-induced psoriasis mice (*n* = 6 per group) treated with 100 mM L-THE or H_2_O for 5 days. Data is representative of three independent experiments. *p* values are determined by two-way ANOVA. ***p < 0.001.

Since hyperproliferation and abnormal differentiation of keratinocytes are major features of psoriasis (Papp et al., 2021a), we detected the proliferation and differentiation of keratinocyte-associated genes by RNA-seq. Studies have shown that the abnormal expression of KRT was associated with hyperproliferation and abnormal differentiation of keratinocytes, such as KRT6, KRT17 and KRT10 (Zhang et al., 2019b; Xiao et al., 2020). Therefore, we analyzed the expression of KRT genes from the RNA-seq data and found that L-THE could significantly downregulate the mRNA expression of *KRT16*, *KRT17*, *KRT6A* and *KRT6B* which have been reported to associate with the proliferation of keratinocytes (Zhang et al., 2019b) (Figure 2A). The levels of *KRT1* and *KRT10* mRNA, differentiation markers of keratinocytes, were increased after treatment with L-THE in the back skin subjected with IMQ cream (Figure 2A). The volcano plot showed that there were 36 differentially expressed KRT genes, among which, 8 genes were up-regulated such as *KRT1* and *KRT10*, and 28 genes were down-regulated such as *KRT17* and *KRT6A* (Figure 2B). The FPKM value of differentially expressed KRT genes was shown in Supplementary Table S2.

To further confirm the results of RNA-seq, we took advantage of RT-PCR analysis to detect the mRNA levels of *KRT1*, *KRT10*, *KRT17* and *KRT6A*. We found that *KRT1* and *KRT10* mRNA levels were significantly upregulated, while the mRNA levels of *KRT6A* and *KRT17* were markedly decreased after treatment with L-THE in the back skin of mice subjected with IMQ cream (Figure 2C). Immunohistochemical analysis showed an increased level of KRT 10 and a decreased level of KRT6A in ear or back skin from IMQ cream-treated mice after L-THE treatment (Figures 2D,E). Furthermore, the proliferation marker Ki67 was significantly decreased after treatment with L-THE in the ear or back skin of IMQ-induced psoriasis mice (Figure 2F). Therefore, our results showed that L-THE regulates the expression of genes related to proliferation and differentiation of keratinocytes.

### L-THE Downregulates the Expression of Inflammatory Genes

**FIGURE 3:**
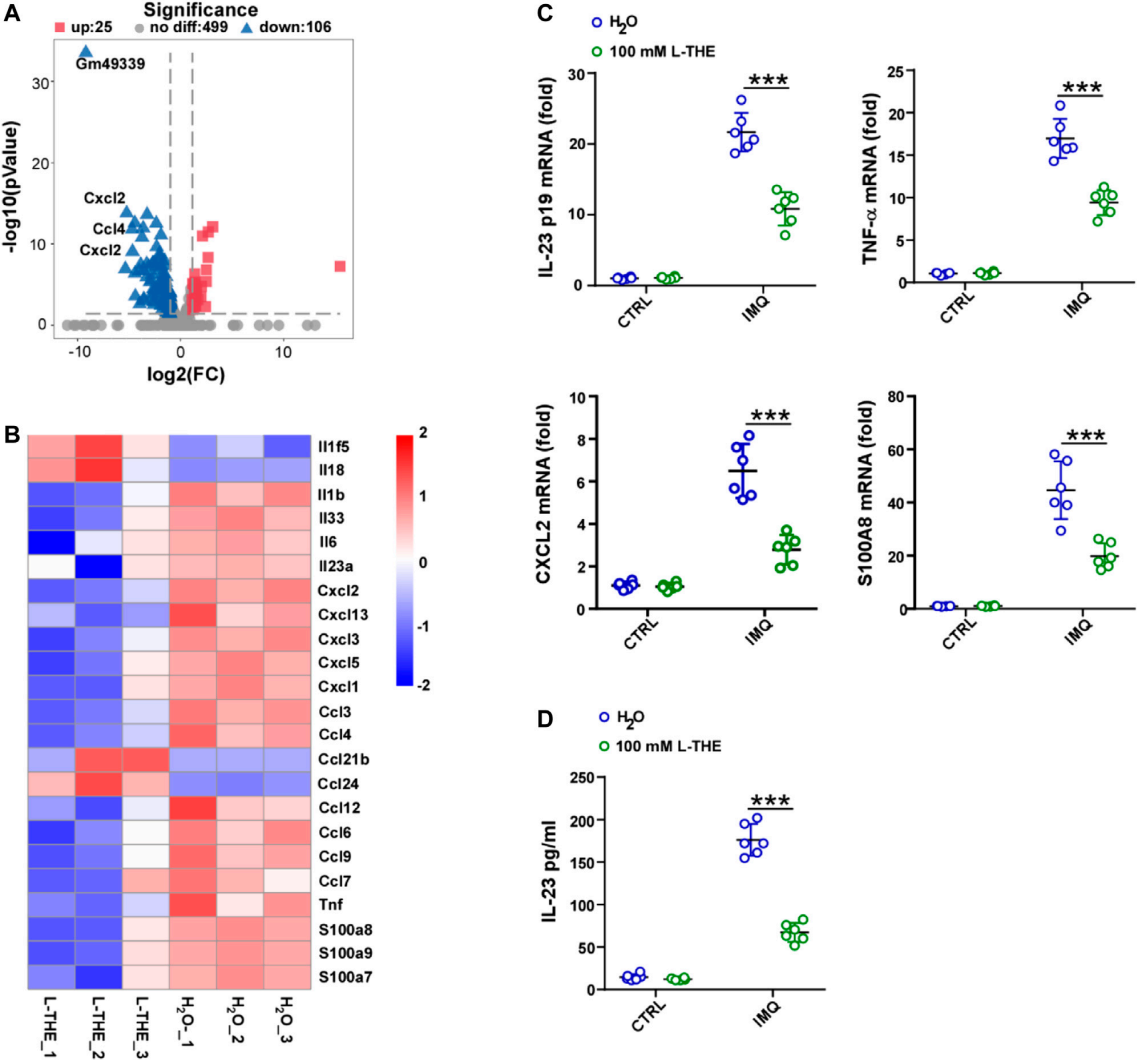
L-THE downregulates the expression of inflammatory genes. **(A,B)** Volcano plot. **(A)** showed the expression of inflammatory response associated genes, and heatmap. **(B)** showed the significantly differentiated genes about interleukin, CXCL, and CCL family genes based on RNA-seq data from IMQ-induced psoriasis mice treated with 100 mM L-THE or H_2_O for 5 days. **(C)** RT-PCR analysis of the mRNA levels of IL-23p19, TNF-α, CXCL2, and S100A8 in IMQ-induced psoriasis mice (*n* = 6 per group) treated with 100 mM L-THE or H_2_O for 0 or 5 days. **(D)** ELISA analysis of IL-23A in IMQ-induced psoriasis mice (*n* = 6 per group) treated with 100 mM L-THE or H2O for 0 or 5 days. Data is representative of three independent experiments. *p* values are determined by two-way ANOVA. ***p < 0.001.

Inflammation is thought to play a critical role in psoriasis (Kaiser et al., 2021). Next, we analyzed the inflammatory response associated genes by gene ontology (GO) analysis which functionally annotated and classified differentially expressed genes. We found 106 genes were down-regulated, while 25 genes were up-regulated (Figure 3A). Among 131 genes, we investigated the differentially expressed genes about interleukin (IL), chemokine (C-X-C motif) ligand (CXCL) chemokine family, C-C motif (CCL) ligand chemokine family and found that the mRNA levels of *IL-1β, IL-33, IL-6* and *IL-23A* were significantly decreased after L-THE treatment (Figure 3B). The mRNA levels of chemokines such as *CXCL1-3, CCL3-4* were downregulated in back skin from IMQ-induced psoriasis mice after L-THE treatment (Figure 3B). We also found that L-THE decreased the mRNA levels of inflammatory cytokines such as *TNF-α, S100A7-9* (Figure 3B). The FPKM value of differentially expressed inflammatory genes was shown in Supplementary Table S3. RT-PCR analysis showed that the mRNA levels of IL-23 P19, TNF-α, CXCL2 and S100A8 were significantly attenuated in back skin from IMQ-induced psoriasis mice (Figure 3C). The protein level of IL-23A was significantly decreased in back skin from IMQ-induced psoriasis mice (Figure 3D). Our results indicated that L-THE downregulates the expression of inflammatory cytokine such as IL-23 and CXCL1.

### L-THE Regulates the Gene Expression Profiles of Extracellular Space, Cell Surface and Extracellular Region

**FIGURE 4:**
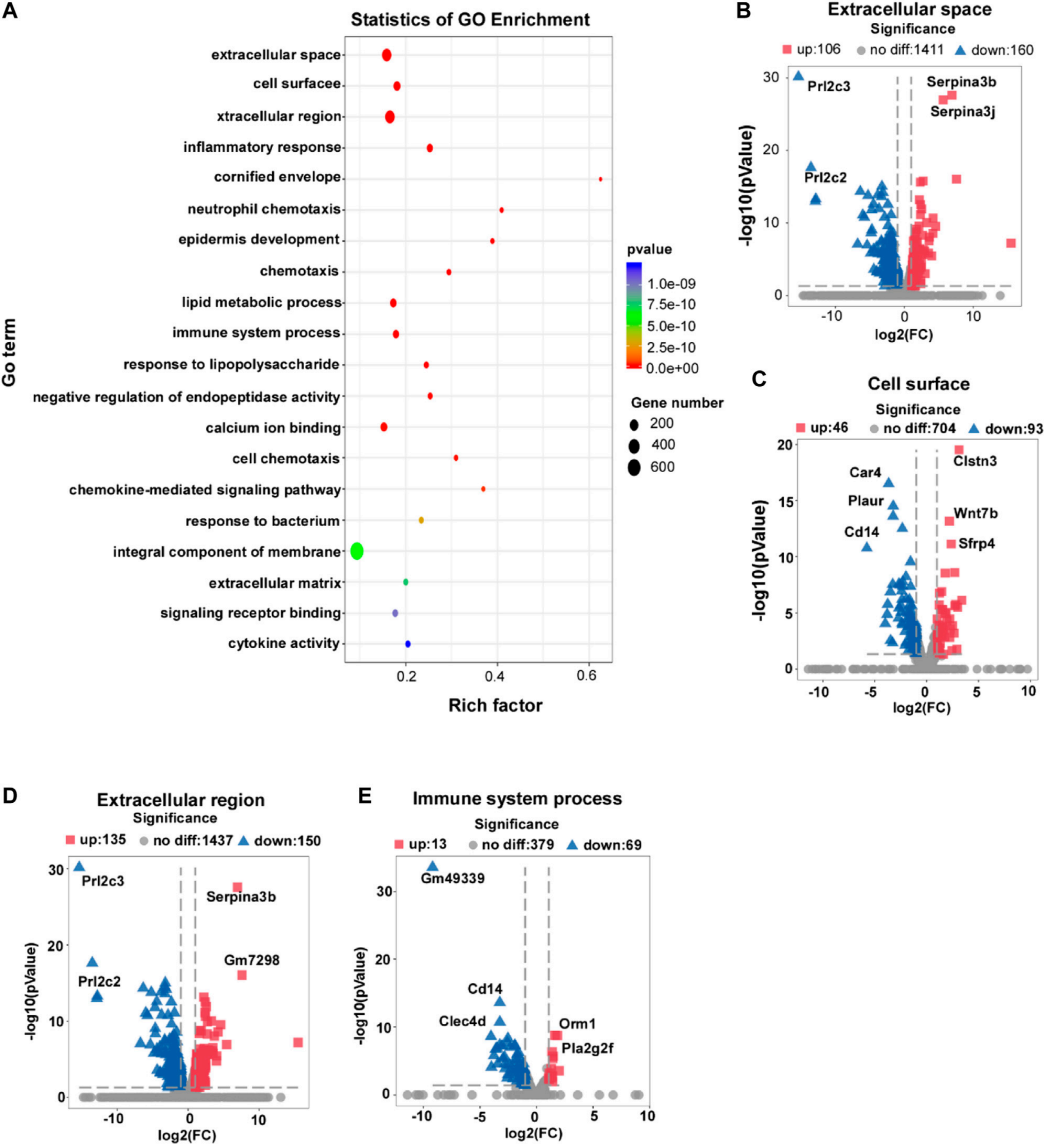
L-THE regulates the gene expression profiles of extracellular space, cell surface and extracellular region. **(A)** Scatter plot for GO analysis based on RNA-seq data from IMQ-induced psoriasis mice treated with 100 mM L-THE or H2O for 5 days. **(B–E)** Volcano plot showed the expression of extracellular space **(B)**, cell surface **(C)**, extracellular region **(D)**, and immune system process. **(E)** associated genes.

Next, we investigated the gene expression profiles regulated by L-THE in the skin from IMQ-induced psoriasis mice by GO analysis. We found gene expression profiles of extracellular space, cell surface and extracellular region were most significantly enriched (Figure 4A). 106 genes were up-regulated, while 160 genes were down-regulated by L-THE in the gene expression profile of extracellular space. Among which, the expression changes of *Serpina3b*, *Serpina3j*, *Prl2c3* and *Prl2c2* were the most significant after L-THE treatment (Figure 4B). L-THE treatment up-regulated 46 genes and down-regulated 93 genes in the gene expression profile of cell surface associated genes, among which, *Clstn3*, *Wnt7b* and *Sfrp4* were the most prominently increased genes, while *Car4*, *Plaur* and *Cd14* were the most significantly decreased genes (Figure 4C). Furthermore, L-THE treatment significantly increased the expression of 135 genes and dramatically suppressed the expression of 150 genes associated with extracellular region (Figure 4D). L-THE treatment also up-regulated 13 genes and down-regulated 69 genes in the gene expression profile of immune system process, among which, *Cd14*, *Clec4d*, *Orm1* and *Pla2g2f* were the most significantly changed genes (Figure 4E). The FPKM value of differentially expressed extracellular space, cell surface, extracellular region, and immune system process associated genes are shown in Supplementary Tables S4–S7. Our results showed that L-THE regulates the gene expression profile of extracellular space, cell surface and extracellular region.

### L-THE Treatment Regulates the Expression of Cytokine-Cytokine Receptor Interaction, Chemokine Signaling Pathway, and IL-17A Signaling Pathway Associated Genes

**FIGURE 5:**
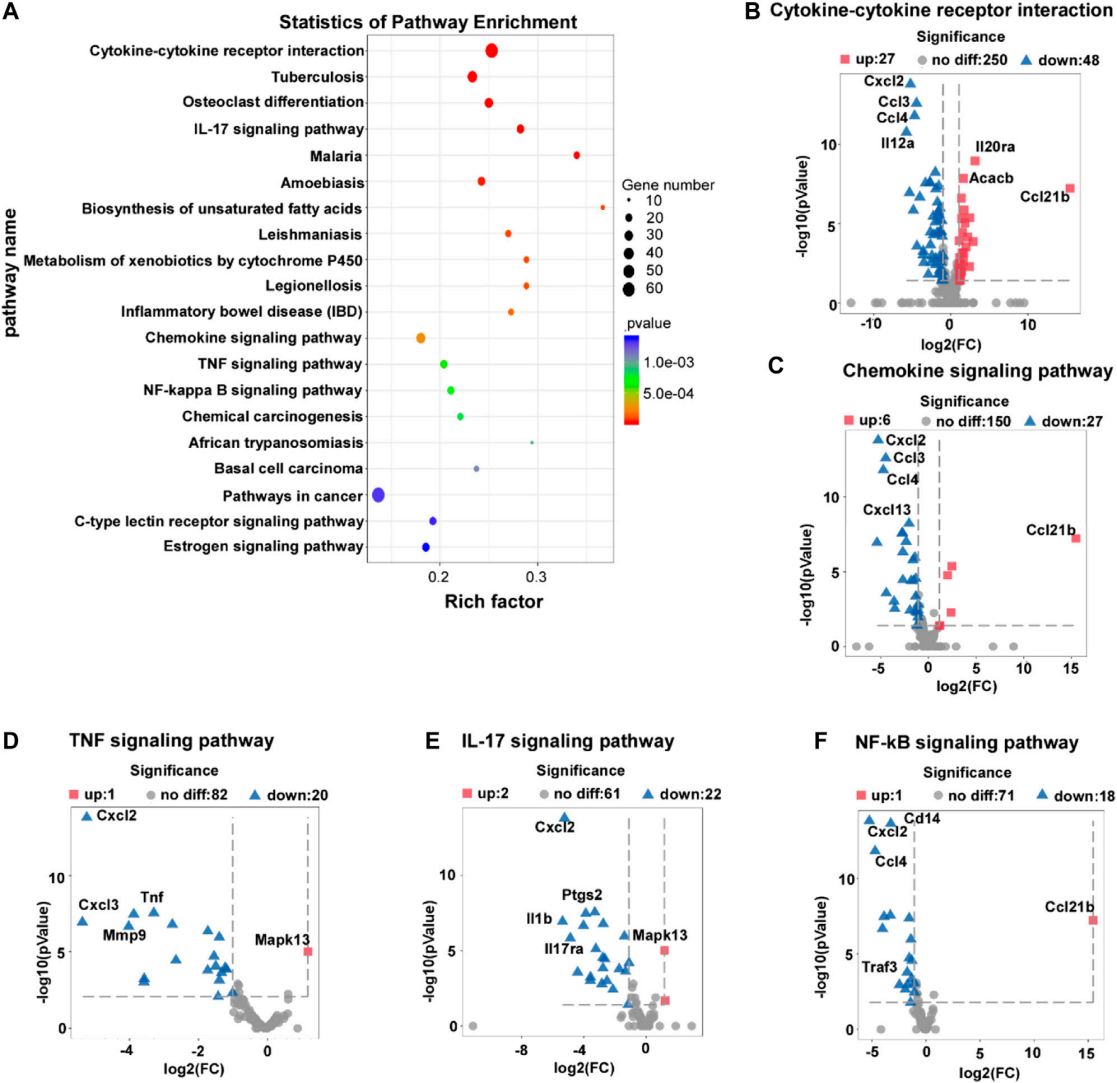
L-THE treatment regulates the expression of cytokine-cytokine receptor interaction, chemokine signaling pathway, and IL-17A signaling pathway associated genes. **(A)** Scatter plot for KEGG analysis based on RNA-seq data from IMQ-induced psoriasis mice treated with 100 mM L-THE or H2O for 5 days. **(B–F)** Volcano plot showed the expression of cytokine-cytokine receptor interaction **(B)**, chemokine signaling pathway **(C)**, TNF signaling pathway **(D)**, IL-17A signaling pathway **(E)**, and NF-κB signaling pathway associated genes.

To investigate the mechanisms of L-THE on IMQ-induced psoriasis in mice, we performed the KEGG pathway enrichment analysis and found that L-THE mainly affected the gene expression profiles of cytokine-cytokine receptor interaction, chemokine signaling pathway, and IL-17A signaling pathway (Figure 5A). L-THE treatment increased the expression of 27 genes and significantly decreased the expression of 48 genes associated with cytokine-cytokine receptor interaction, among which, *CXCL2, CCL3* and *CCL4* were the most prominently decreased genes (Figure 5B). 6 genes were up-regulated, while 27 genes were down-regulated by L-THE in the gene expression profile of chemokine signaling pathway (Figure 5C). The expression of *CXCL2, CCL3* and *CCL4* were also the most prominently decreased genes (Figure 5C). L-THE regulated the TNF signaling pathway associated genes, among which 20 genes were downregulated such as *TNF*, and 1 gene was increased (Figure 5D). Furthermore, the gene expression profile of *IL-17A* signaling pathway was significantly changed by L-THE, among which, *IL-1β* and *IL-17RA* expression were downregulated (Figure 5E). We also found L-THE treatment regulated the gene expression profile of *NF-κB* signaling pathway, among which, the expression of *CXCL3* and *CD14* were significantly decreased after L-THE treatment (Figure 5F). The heatmap also showed L-THE regulated the expression of cytokine-cytokine receptor interaction, chemokine, TNF, NF-κB and IL-17A signaling pathway associated genes (Supplementary Figure S1). The FPKM value of differentially expressed cytokine-cytokine receptor interaction, chemokine TNF, NF-κB and IL-17A signaling pathway associated genes was shown in Supplementary Tables S8–S11. Our results indicated that L-THE treatment mainly affects the expression of cytokine-cytokine receptor interaction, chemokine signaling pathway, and *IL-17A* signaling pathway associated genes.

### L-THE Upregulates the Propanoate Metabolism by Increasing the Expression of Propanoate Metabolism Associated Genes

**FIGURE 6:**
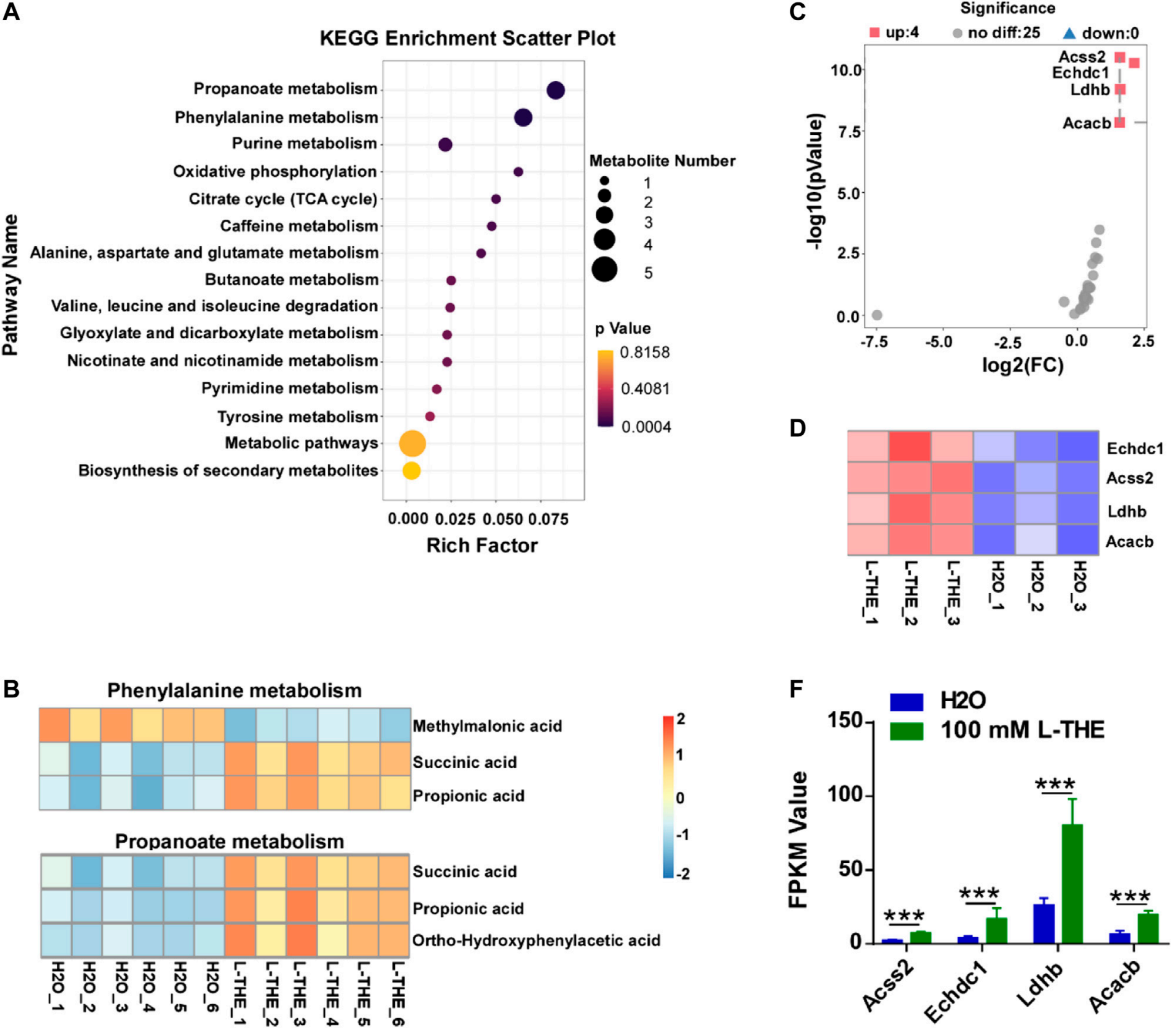
L-THE upregulates the propanoate metabolism by increasing the expression of propanoate metabolism associated genes. **(A)** Scatter plot for KEGG analysis based on untargeted metabolism data from IMQ-induced psoriasis mice treated with 100 mM L-THE or H2O for 5 days. **(B)** Heatmap showed the significantly changed production of phenylalanine and propanoate metabolism based on untargeted metabolism data from IMQ-induced psoriasis mice treated with 100 mM L-THE or H2O for 5 days. **(C,D)** Volcano plot. **(C)** showed the expression of propanoate metabolism associated genes, and heatmap. **(D)** showed the significantly differentiated propanoate metabolism genes based on RNA-seq data from IMQ-induced psoriasis mice treated with 100 mM L-THE or H_2_O for 5 days. **(E)** The FPKM Value of Acss2, Echdc1, Ldhb, and Acacb in **(D)**.

Metabolic abnormalities have been reported to associate with the development of psoriasis (Cao et al., 2021). Therefore, we detected the metabolic changes in the back skin from mice with psoriasis after L-THE treatment by untargeted metabolomics. We found that L-THE mainly affected the propanoate, and phenylalanine by KEGG enrichment analysis (Figure 6A). The data of KEGG enrichment analysis in metabolic pathway was showed in Supplementary Table S12. Next, we analyzed the significantly changed metabolites of propanoate, and phenylalanine purine metabolism after L-THE treatment. The metabolites of propanoate metabolism including succinic acid, propionic acid, ortho-hydroxyphenylacetic acid were significantly downregulated in back skin from mice with psoriasis after L-THE treatment (Figure 6B). However, the significantly changed metabolites of phenylalanine, and purine metabolism regulated by L-THE were inconsistent (Figure 6B). So, we focused on the propanoate metabolism and investigated the expression of propanoate metabolism associated genes by RNA-seq analysis. 4 genes were upregulated in the gene expression profile of propanoate metabolism after L-THE treatment, such as *Acss2, Echdc1, Ldhb,* and *Acacb* (Figures 6C,D). Therefore, our results demonstrated that L-THE promotes propanoate metabolism by increasing the expression of propanoate metabolism associated genes.

### L-THE Inhibits the Expression of IL-23 and Chemokines *in vitro*

**FIGURE 7:**
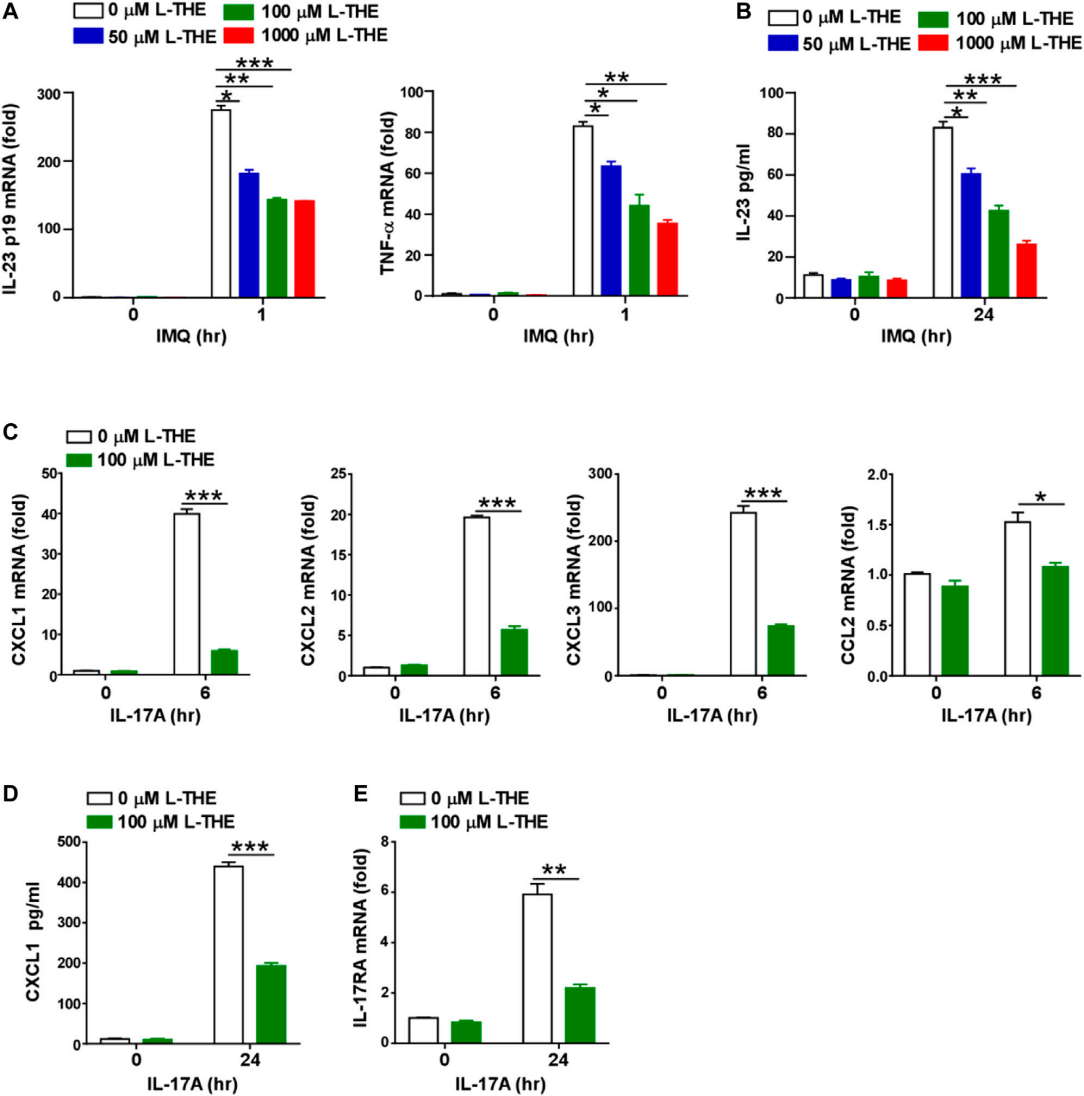
L-THE inhibites the expression of IL-23 and chemokines *in vitro*. **(A)** Q-PCR analysis of IL-23p19 and TNF-α in mRNA in BMDCs were treated with 4 μg/ml IMQ for indicated time points after pretreatment with L-THE for 12 h. **(B)** ELISA analysis of IL-23 protein in BMDCs were treated with 4 μg/ml IMQ for indicated time points after pretreatment with L-THE for 12 h. **(C)** Q-PCR analysis of CXCL1-3, and CCL2 mRNA in mouse primary keratinocytes were treated with 100 ng/ml IL-17A for indicated time points after pretreatment with L-THE for 12 h. **(D)** ELISA analysis of CXCL1 protein in mouse primary keratinocytes were treated with 100 ng/ml IL-17A for indicated time points after pretreatment with L-THE for 12 h. **(E)** Q-PCR analysis of IL-17RA mRNA in mouse primary keratinocytes were treated with 100 ng/ml IL-17A for indicated time points after pretreatment with L-THE for 12 h. Data is representative of three independent experiments. *p* values are determined by two-way ANOVA. *, *p* < 0.05; **, *p* < 0.01; ***, *p* < 0.001.

In the above results, we found that L-THE downregulated the expression of IL-23 in skin tissues from mice with psoriasis. Due to IL-23 is mainly produced by dendritic cell during the development of psoriasis, we investigated whether L-THE could regulate the expression of IL-23 in BMDCs. The mRNA levels of *IL-23A* and *TNF-α* were significantly downregulated in L-THE treated DCs after IMQ stimulation (Figure 7A). ELISA assay showed that the production of IL-23A was decreased in BMDCs pre-treated with L-THE (Figure 7B).

We had found L-THE regulated chemokine signaling pathway and IL-17A signaling pathway associated genes, such as *CXCL2, CCL3* and *CCL4* in skin tissues from mice with psoriasis (Figure 5). Studies have shown that chemokines are also produced from keratinocytes after stimulation with IL-17A (Nedoszytko et al., 2014). So, we detected whether L-THE could regulate the expression of chemokines in mouse primary keratinocytes treated with IL-17A. We found that the mRNA levels of *CXCL1-3* and *CCL2* were significantly downregulated in keratinocytes pre-treated with L-THE after IL-17A stimulation (Figure 7C). We found the protein level of CXCL1 was also decreased in keratinocytes pre-treated with L-THE after IL-17A stimulation (Figure 7D). Furthermore, the mRNA level of IL-17RA was lower than control in keratinocytes pre-treated with L-THE (Figure 7E). Our results demonstrated that L-THE inhibits the expression of IL-23 in DCs, and downregulates the production of chemokines in keratinocytes by decreasing the expression of IL-17RA.

## Discussion

Psoriasis is a common inflammatory skin disease characterized by aberrant inflammation and epidermal hyperplasia (Peinemann et al., 2021). Drugs that systemically inhibit TNF-α, IL-17 and IL-23 cytokines such as infliximab, secukinumab, and ustekinumab have been proved to be effective and safe therapeutic agents for the treatment of psoriasis (Puig, 2017; Papp et al., 2021b). However, it is common for psoriasis to recur in previously inflamed areas. Therefore, it is crucial to prevent the recurrence of psoriasis after treatment, and inhibition of inflammatory response is an effective strategy. L-THE, a natural constituent in tea, has been granted GRAS (generally recognized as safe) status by the Food and Drug Administration (FDA) and has influential effects on lifestyle associated diseases, such as stress, cardiovascular disorders, diabetes, and hypertension (Shojaei-Zarghani et al., 2021). Here, we found that topical applied L-THE significantly alleviated the inflammation of IMQ-induced psoriasis like skin in mice by decreasing the inflammatory response. Emerging evidences display that there are no significant adverse effects related to excessive L‐THE intake (Unno et al., 2020a; Unno et al., 2020b). Therefore, we speculated and suggested that L-THE can be used as an adjuvant topical treatment to prevent the relapse of psoriasis in clinical.

L‐THE is a nonproteinogenic amino acid derived from tea (*Camellia sinensis*), and exhibits strong antioxidant and anti‐inflammatory effects (Zhang et al., 2019a; Xu et al., 2020). A large number of studies have been revealed that L-THE significantly improves the neuroprotection, stress‐related disorders, sleep quality, and cognition (Matsuu-Matsuyama et al., 2020; Paiva et al., 2020; Williams et al., 2020). It has been reported that L-THE has an effective treatment for alternative anxiety, stress, and depression, which provides a variety of psychological benefits (Williams et al., 2019). Furthermore, L‐THE has been shown to promote the effectivity of antitumor drugs such as adriamycin, and idarubicin (Shojaei-Zarghani et al., 2021). L‐THE can also regulate the innate and adaptive immunity (Zhang et al., 2019a). For example, coadministration of L‐THE and cysteine displays lower viral titers in lung and increases the levels of anti-DNP, anti-IgG and anti-IgM in serum after influenza virus infection (Takagi et al., 2010). L-THE treatment significantly prevent the CCL4-induced hepatic cirrhosis through decreasing the proinflammatory response and profibrogenic signal pathway (Pérez-Vargas et al., 2016). L-THE has been reported to ameliorate the 12-O-tetradecanoylphorbol-13-acetate-induced acute skin inflammation (Zeng et al., 2018). In this study, we found the function and mechanism of L-THE on psoriasis in psoriasis-like mouse model induced by IMQ.

IMQ is a ligand for Toll-like receptors of macrophages, monocytes, and plasmacytoid dendritic cells (pDCs), and initiates the inflammatory response. IMQ-treated mouse skin closely resembles human plaque-type psoriasis and accompanies with skin thickening, erythema, scaling (van der Fits et al., 2009; Bochenska et al., 2017). In addition, it has been demonstrated that IL-23/IL-17 axis is very crucial in the process of psoriasis (van der Fits et al., 2009). In our study, we found that L-THE alleviated epidermal thickness and inflammatory response in mice with IMQ-induced psoriasis like skin inflammation. Furthermore, the expression of proliferation and differentiation of keratinocytes associated genes were regulated, and the expression of inflammatory genes was significantly decreased in psoriasis mice after treatment with L-THE. The RNA-Seq analysis showed that L-THE significantly decreased the production of IL-23 in dendritic cells after IMQ treatment *in vitro*. Moreover, the production of chemokines such as CXCL1-3, and CCL2 was markedly inhibited in keratinocytes upon IL-17A stimulation by downregulating the expression of IL-17RA. Therefore, our study provided a new insight on the function of L-THE in inflammatory disease.

Psoriasis is a chronic inflammatory condition and mainly affects skin and joints. However, psoriasis is also a systemic disease that is associated with a range of comorbidities, especially metabolic syndrome (MetS) and cardiovascular disease (Singh et al., 2017). MetS is defined as a pathological condition accompanied by abdominal obesity, dyslipidemia, insulin resistance, and hypertension, and is an important risk contributor to multiple chronic diseases, such as cardiovascular disease, diabetes mellitus. It is important to early recognize MetS and exerts effective interventions (Hu et al., 2019). L-THE exerts its influential effects on stress‐related disorders, diabetes, cardiovascular disorders, hypertension and liver injury through multiple mechanisms (Katasonov, 2018). L-THE increases serotonin, dopamine, GABA, and glycine levels in various areas of the brain to improve relaxation, cognition and sleep quality (Kim et al., 2019). The neuroprotective mechanisms of L-THE include the reduction of oxidative protein and lipid damage as well as the elevation of GSH levels to prevent neurotoxicity and environmental toxin‐induced neuronal cell death in the brain (Matsuu-Matsuyama et al., 2020; Saeed et al., 2020). It has been reported that L-THE regulates glucose, lipid, and protein metabolism by promoting the activation of insulin and AMP-activated kinase signaling pathways (Peng et al., 2021). L-THE can decrease the expression of IL-1β, IL-6 and TGF-β though inhibiting the activation of NF‐κB signaling pathway to prevents liver fibrosis (Pérez-Vargas et al., 2016). Here, we found that L-THE significantly decreased the production of IL-23 in dendritic cells after IMQ treatment *in vitro*. Moreover, the production of chemokines such as CXCL1-3, and CCL2 was markedly inhibited in keratinocytes upon IL-17A stimulation by downregulated the expression of IL-17RA. KEGG pathway analysis revealed that L-THE regulated the activation of the NF‐κB and IL-17A signaling pathway. Furthermore, we found that L-THE promoted the propanoate metabolism which is typically arises from the production of the acid by gut or skin microflora and has been reported to protect from hypertensive cardiovascular damage by inhibiting the activity of TH17 (Bartolomaeus et al., 2019). Therefore, our results suggested that L-THE downregulates the production of IL-23 and chemokines, and attenuates the IMQ-induced psoriasis like skin inflammation by inhibiting the activation of NF‐κB and IL-17A signaling pathway, and promotes the propanoate metabolism. Further study needs to confirm whether L-THE can respectively restrain the activation of NF‐κB, IL-17A signaling pathway, and propanoate metabolism in dendritic cells or keratinocytes.

## Conclusion

In summary, our study found a new insight on the function of L-THE in inflammatory disease. Specifically, L-THE can significantly decrease the levels of IL-23 and chemokines, and attenuate the IMQ-induced psoriasis like skin inflammation by inhibiting the activation of NF‐κB and IL-17A signaling pathway. Therefore, we suggested that L-THE can be used as a potential treatment for psoriasis, or as an adjuvant treatment of ustekinumab or secukinumab to prevent the relapse of psoriasis in clinical.

## Data Availability

The data presented in this study are deposited in the Sequence Read Archive repository, accession number (PRJNA742565). The datasets presented in this study can be found in online repositories.

## References

[B1] Bartolomaeus H. Balogh A. Yakoub M. Homann S. Markó L. Höges S. (2019). Short-Chain Fatty Acid Propionate Protects from Hypertensive Cardiovascular Damage. Circulation 139, 1407–1421. 10.1161/circulationaha.118.036652 30586752 PMC6416008

[B2] Bochenska K. Smolinska E. Moskot M. Jakobkiewicz-Banecka J. Gabig-Ciminska M. (2017). Models in the Research Process of Psoriasis. Int. J. Mol. Sci. 18, 2514. 10.3390/ijms18122514 PMC5751117 29186769

[B3] Boutet M. A. Nerviani A. Gallo Afflitto G. Pitzalis C. (2018). Role of the IL-23/IL-17 Axis in Psoriasis and Psoriatic Arthritis: The Clinical Importance of its Divergence in Skin and Joints. Int. J. Mol. Sci. 19, 530. 10.3390/ijms19020530 PMC5855752 29425183

[B4] Campa M. Mansouri B. Warren R. Menter A. (2016). A Review of Biologic Therapies Targeting IL-23 and IL-17 for Use in Moderate-To-Severe Plaque Psoriasis. Dermatol. Ther. (Heidelb). 6, 1–12. 10.1007/s13555-015-0092-3 26714681 PMC4799039

[B5] Cao H. Su S. Yang Q. Le Y. Chen L. Hu M. (2021). Metabolic Profiling Reveals interleukin-17A Monoclonal Antibody Treatment Ameliorate Lipids Metabolism with the Potentiality to Reduce Cardiovascular Risk in Psoriasis Patients. Lipids Health Dis. 20, 16. 10.1186/s12944-021-01441-9 33602246 PMC7890626

[B6] Damiani G. Pacifico A. Chu S. Ching Chi C. Young Dermatologists Italian Network (2021). Frequency of Phototherapy for Treating Psoriasis: a Systematic Review. Ital. J. Dermatol. Venerol. in press. 10.1111/ced.14631 33982550

[B7] Di Cesare A. Di Meglio P. Nestle F. O. (2009). The IL-23/Th17 axis in the Immunopathogenesis of Psoriasis. J. Invest. Dermatol. 129, 1339–1350. 10.1038/jid.2009.59 19322214

[B8] Egeberg A. Andersen Y. M. F. Halling‐Overgaard A. S. Alinaghi F. Thyssen J. P. Burge R. (2020). Corrigendum: Systematic Review on Rapidity of Onset of Action for Interleukin‐17 and Interleukin‐23 Inhibitors for Psoriasis. J. Eur. Acad. Dermatol. Venereol. 34, 2156. 10.1111/jdv.16827 33448477

[B9] Geller S. Xu H. Lebwohl M. Nardone B. Lacouture M. E. Kheterpal M. (2018). Malignancy Risk and Recurrence with Psoriasis and its Treatments: A Concise Update. Am. J. Clin. Dermatol. 19, 363–375. 10.1007/s40257-017-0337-2 29260411 PMC5948118

[B10] Ghoreschi K. Balato A. Enerbäck C. Sabat R. (2021). Therapeutics Targeting the IL-23 and IL-17 Pathway in Psoriasis. The Lancet. 397, 754–766. 10.1016/s0140-6736(21)00184-7 33515492

[B11] Gonzalez-Cantero A. Ortega-Quijano D. Alvarez-Diaz N. Ballester A. Jimenez-Gomez N. Jaen P. (2021). Impact of Biologic Agents on Imaging and Biomarkers of Cardiovascular Disease in Patients with Psoriasis: a Systematic Review and Meta-Analysis of Randomized Placebo-Controlled Trials. J. Invest. Dermatol. in press, 01149. 10.1016/j.jid.2021.03.024 33891953

[B12] Hu Y. Zhu Y. Lian N. Chen M. Bartke A. Yuan R. (2019). Metabolic Syndrome and Skin Diseases. Front. Endocrinol. (Lausanne). 10, 788. 10.3389/fendo.2019.00788 31824416 PMC6880611

[B13] Kagami S. (2011). IL-23 and Th17 Cells in Infections and Psoriasis. Jpn. J. Clin. Immunol. 34, 13–19. 10.2177/jsci.34.13 21372509

[B14] Kaiser H. Kvist-Hansen A. Krakauer M. Gortz P. M. Henningsen K. M. A. Wang X. (2021). Association between Vascular Inflammation and Inflammation in Adipose Tissue, Spleen, and Bone Marrow in Patients with Psoriasis. Life (Basel) 11, 305. 10.3390/life11040305 33915972 PMC8065955

[B15] Katasonov A. B. (2018). Neurobiological Effects of Theanine and its Possible Use in Neurology and Psychiatry. Z. Nevrol. Psikhiatr. Im. S.S. Korsakova. 118, 118–124. 10.17116/jnevro2018118111118 30585616

[B16] Kim S. Jo K. Hong K. B. Han S. H. Suh H. J. (2019). GABA and L-Theanine Mixture Decreases Sleep Latency and Improves NREM Sleep. Pharm. Biol. 57, 65–73. 10.1080/13880209.2018.1557698 30707852 PMC6366437

[B17] Li R. Song Z. Zhao J. Huo D. Fan Z. Hou D.-X. (2018). Dietary L-Theanine Alleviated Lipopolysaccharide-Induced Immunological Stress in Yellow-Feathered Broilers. Anim. Nutr. 4, 265–272. 10.1016/j.aninu.2018.05.002 30175254 PMC6116832

[B18] Lillis J. V. Guo C. S. Lee J. J. Blauvelt A. (2010). Increased IL-23 Expression in Palmoplantar Psoriasis and Hyperkeratotic Hand Dermatitis. Arch. Dermatol. 146, 918–919. 10.1001/archdermatol.2010.168 20713832

[B19] Martinez-Moreno A. Ocampo-Candiani J. Garza-Rodriguez V. (2020). Psoriasis and Cardiovascular Disease: A Narrative Review. Korean J. Fam. Med. in press. 10.4082/kjfm.20.0053 PMC8490176 32512983

[B20] Matsuu-Matsuyama M. Shichijo K. Tsuchiya T. Kondo H. Miura S. Matsuda K. (2020). Protective Effects of a Cystine and Theanine Mixture against Acute Radiation Injury in Rats. Environ. Toxicol. Pharmacol. 78, 103395. 10.1016/j.etap.2020.103395 32325407

[B21] Nedoszytko B. Sokołowska-Wojdyło M. Ruckemann-Dziurdzińska K. Roszkiewicz J. Nowicki R. J. (2014). Chemokines and Cytokines Network in the Pathogenesis of the Inflammatory Skin Diseases: Atopic Dermatitis, Psoriasis and Skin Mastocytosis. pdia 2, 84–91. 10.5114/pdia.2014.40920 PMC4112246 25097473

[B22] Niculet E. Radaschin D. S. Nastase F. Draganescu M. Baroiu L. Miulescu M. (2020). Influence of Phytochemicals in Induced Psoriasis (Review). Exp. Ther. Med. 20, 3421–3424. 10.3892/etm.2020.9013 32905089 PMC7465111

[B23] No D. J. Amin M. Reddy S. P. Egeberg A. Wu J. J. (2017). Sites of Recurrence in Patients Following Clearance of Psoriasis with Biologic Therapy. J. Eur. Acad. Dermatol. Venereol. 31, e297–e298. 10.1111/jdv.14072 27896876

[B24] Paiva L. Lima E. Motta M. Marcone M. Baptista J. (2020). Variability of Antioxidant Properties, Catechins, Caffeine, L-Theanine and Other Amino Acids in Different Plant Parts of Azorean Camellia Sinensis. Curr. Res. Food Sci. 3, 227–234. 10.1016/j.crfs.2020.07.004 33426532 PMC7782930

[B25] Papp K. A. Gniadecki R. Beecker J. Dutz J. Gooderham M. J. Hong C. H. (2021). Psoriasis Prevalence and Severity by Expert Elicitation. Dermatol. Ther. (Heidelb). 11, 1053–1064. 10.1007/s13555-021-00518-8 33886086 PMC8163919

[B26] Papp K. A. Weinberg M. A. Morris A. Reich K. (2021). IL17A/F Nanobody Sonelokimab in Patients with Plaque Psoriasis: a Multicentre, Randomised, Placebo-Controlled, Phase 2b Study. The Lancet. 397, 1564–1575. 10.1016/s0140-6736(21)00440-2 33894834

[B27] Peinemann F. Harari M. Peternel S. Chan T. Chan D. Labeit A. M. (2021). Indoor Balneophototherapy for Chronic Plaque Psoriasis: Abridged Cochrane Review. Dermatol. Ther. 34, e14588. 10.1111/dth.14588 33236826

[B28] Peng W. Q. Xiao G. Li B. Y. Guo Y. Y. Guo L. Tang Q. Q. (2021). L-theanine Activates the Browning of White Adipose Tissue through the AMPK/alpha-Ketoglutarate/Prdm16 Axis and Ameliorates Diet-Induced Obesity in Mice. Diabetes 70, 1458–1472. 10.2337/db20-1210 33863801

[B29] Pérez-Vargas J. Zarco N. Vergara P. Shibayama M. Segovia J. Tsutsumi V. (2016). l-Theanine Prevents Carbon Tetrachloride-Induced Liver Fibrosis via Inhibition of Nuclear Factor κB and Down-Regulation of Transforming Growth Factor β and Connective Tissue Growth Factor. Hum. Exp. Toxicol. 35, 135–146. 10.1177/0960327115578864 25852135

[B30] Puig L. (2017). The Role of IL 23 in the Treatment of Psoriasis. Expert Rev. Clin. Immunol. 13, 525–534. 10.1080/1744666x.2017.1292137 28165883

[B31] Saeed M. Khan M. S. Kamboh A. A. Alagawany M. Khafaga A. F. Noreldin A. E. (2020). L-theanine: an Astounding Sui Generis Amino Acid in Poultry Nutrition. Poult. Sci. 99, 5625–5636. 10.1016/j.psj.2020.07.016 33142480 PMC7647716

[B32] Sharma E. Joshi R. Gulati A. (2018). l-Theanine: An Astounding Sui Generis Integrant in tea. Food Chem. 242, 601–610. 10.1016/j.foodchem.2017.09.046 29037735

[B33] Shojaei-Zarghani S. Rafraf M. Yari-Khosroushahi A. (2021). Theanine and Cancer: A Systematic Review of the Literature. Phytother Res. in press. 10.1002/ptr.7110 33891786

[B34] Singh S. Young P. Armstrong A. W. (2017). An Update on Psoriasis and Metabolic Syndrome: A Meta-Analysis of Observational Studies. PLoS One. 12, e0181039. 10.1371/journal.pone.0181039 28719618 PMC5515416

[B35] Takagi Y. Kurihara S. Higashi N. Morikawa S. Kase T. Maeda A. (2010). Combined Administration L-Cystine and L-Theanine Enhances Immune Functions and Protects against Influenza Virus Infection in Aged Mice. J. Vet. Med. Sci. 72, 157–165. 10.1292/jvms.09-0067 19940390

[B36] Unno K. Muguruma Y. Inoue K. Konishi T. Taguchi K. Hasegawa-Ishii S. (2020). Theanine, Antistress Amino Acid in Tea Leaves, Causes Hippocampal Metabolic Changes and Antidepressant Effects in Stress-Loaded Mice. Int. J. Mol. Sci. 22, 193. 10.3390/ijms22010193 PMC7795947 33379343

[B37] Unno K. Sumiyoshi A. Konishi T. Hayashi M. Taguchi K. Muguruma Y. (2020). Theanine, the Main Amino Acid in Tea, Prevents Stress-Induced Brain Atrophy by Modifying Early Stress Responses. Nutrients 12, 174. 10.3390/nu12010174 PMC7019546 31936294

[B38] van der Fits L. Mourits S. Voerman J. S. A. Kant M. Boon L. Laman J. D. (2009). Imiquimod-induced Psoriasis-like Skin Inflammation in Mice Is Mediated via the IL-23/IL-17 axis. J. Immunol. 182, 5836–5845. 10.4049/jimmunol.0802999 19380832

[B39] Wang D. Gao Q. Zhao G. Kan Z. Wang X. Wang H. (2018). Protective Effect and Mechanism of Theanine on Lipopolysaccharide-Induced Inflammation and Acute Liver Injury in Mice. J. Agric. Food Chem. 66, 7674–7683. 10.1021/acs.jafc.8b02293 29969892

[B40] Wang Q. Zhang X. Lei S. Wang Y. Zhuang Y. Chen Y. (2018). RNA Sequence Analysis Reveals Pathways and Candidate Genes Associated with Liver Injury in a Rat Pancreatitis Model. Pancreatology 18, 753–763. 10.1016/j.pan.2018.08.006 30150111

[B41] Williams J. L. Everett J. M. D’Cunha N. M. Sergi D. Georgousopoulou E. N. Keegan R. J. (2020). The Effects of Green Tea Amino Acid L-Theanine Consumption on the Ability to Manage Stress and Anxiety Levels: a Systematic Review. Plant Foods Hum. Nutr. 75, 12–23. 10.1007/s11130-019-00771-5 31758301

[B42] Williams J. Sergi D. McKune A. J. Georgousopoulou E. N. Mellor D. D. Naumovski N. (2019). The Beneficial Health Effects of green tea Amino Acid L -theanine in Animal Models: Promises and Prospects for Human Trials. Phytotherapy Res. 33, 571–583. 10.1002/ptr.6277 30632212

[B43] Xiao C.-Y. Zhu Z.-L. Zhang C. Fu M. Qiao H.-J. Wang G. (2020). Small Interfering RNA Targeting of Keratin 17 Reduces Inflammation in Imiquimod-Induced Psoriasis-like Dermatitis. Chin. Med. J. (Engl). 133, 2910–2918. 10.1097/cm9.0000000000001197 33237695 PMC7752698

[B44] Xu W. Lin L. Liu A. Zhang T. Zhang S. Li Y. (2020). L-theanine Affects Intestinal Mucosal Immunity by Regulating Short-Chain Fatty Acid Metabolism under Dietary Fiber Feeding. Food Funct. 11, 8369–8379. 10.1039/d0fo01069c 32935679

[B45] Yamanaka K. Yamamoto O. Honda T. (2021). Pathophysiology of Psoriasis: A Review. J. Dermatol. 48, 722–731. 10.1111/1346-8138.15913 33886133

[B46] Zeng W.-J. Tan Z. Lai X.-F. Xu Y.-N. Mai C.-L. Zhang J. (2018). Topical Delivery of L -theanine Ameliorates TPA-Induced Acute Skin Inflammation via Downregulating Endothelial PECAM-1 and Neutrophil Infiltration and Activation. Chem. Biol. Interact. 284, 69–79. 10.1016/j.cbi.2018.02.019 29458014

[B47] Zhang C. Chen K. K. Zhao X. H. Wang C. Geng Z. Y. (2019). Effect of L-Theanine on the Growth Performance, Immune Function, and Jejunum Morphology and Antioxidant Status of Ducks. Animal 13, 1145–1153. 10.1017/s1751731118002884 30376911

[B48] Zhang X. Yin M. Zhang L. J. (2019). Keratin 6, 16 and 17-Critical Barrier Alarmin Molecules in Skin Wounds and Psoriasis. Cells 8, 807. 10.3390/cells8080807 PMC6721482 31374826

