# Supplementary material for: L-Theanine Alleviates IMQ-Induced Psoriasis Like Skin Inflammation by Downregulating the Production of IL-23 and Chemokines
Source: Front Pharmacol. 2021 Jul 26;12:719842. doi: 10.3389/fphar.2021.719842 (PMC8350042; doi:10.3389/fphar.2021.719842)
Supplement: Supplementary file 1 [file DataSheet2.docx]

Supplemental tables

Supplemental table 1. The primer sequence for RT-PCR

| Gene name | Forward | Reverse |
| --- | --- | --- |
| GAPDH | AACTTTGGCATTGTGGAAGG | ACACATTGGGGGTAGGAACA |
| IL-23A | AATAATGTGCCCCGTATCCAGT | GCTCCCCTTTGAAGATGTCAG |
| TNF-α | TCCAGTGTGTCCTTCCGAAGT | TGCCTCCGCCAGAACTGTA |
| S100A8 | AAATCACCATGCCCTCTACAAG | CCCACTTTTATCACCATCGCAA |
| CXCL1 | CTGGGATTCACCTCAAGAACATC | CAGGGTCAAGGCAAGCCTC |
| CXCL2 | ATGCCTCACTCGTACCCAG | TTTCCACCCCAATTTGGCTCA |
| CXCL3 | ATGGACAGCAACCATCAAAGTA | GGCTCTGAGTGGCATAGGATAC |
| CCL2 | TTAAAAACCTGGATCGGAACCAA | GCATTAGCTTCAGATTTACGGGT |
| KRT1 | GACACCACAACCCGGACCCAAAACTTAG | ATACTGGGCCTTGACTTCCGAGATGATG |
| KRT10 | GGAGGGTAAAATCAAGGAGTGGTA Krt10 | TCAATCTGCAGCAGCACGTT |
| KRT17 | ACCATCCGCCAGTTTACCTC | CTACCCAGGCCACTAGCTGA |
| KRT6A | AGAGAGGGGTCGCATGAACT | TCATCTGTTAGACTGTCTGCCTT |
| IL-17RA | AGTGTTTCCTCTACCCAGCAC | GAAAACCGCCACCGCTTAC |

Supplemental table 2. The FPKM values of KRT genes.

| gene_name | FPKM.IMQ5_LTH_1 | FPKM.IMQ5_LTH_2 | FPKM.IMQ5_LTH_3 | FPKM.IMQ5_H2O_1 | FPKM.IMQ5_H2O_2 | FPKM.IMQ5_H2O_3 |
| --- | --- | --- | --- | --- | --- | --- |
| Krt10 | 832.01 | 2235.07 | 3505.79 | 243.64 | 158.92 | 104.15 |
| Krt79 | 133.78 | 161.12 | 137.60 | 41.86 | 17.52 | 17.48 |
| Krt1 | 886.18 | 1677.94 | 2553.06 | 299.70 | 183.25 | 106.30 |
| Krt24 | 1.66 | 3.38 | 4.12 | 0.28 | 0.18 | 0.23 |
| Krt15 | 112.34 | 385.13 | 323.72 | 51.09 | 14.19 | 31.37 |
| Krt77 | 2.92 | 19.78 | 44.61 | 1.34 | 0.69 | 0.17 |
| Krt78 | 13.67 | 27.00 | 38.97 | 6.08 | 7.97 | 7.74 |
| Krt42 | 0.29 | 0.22 | 0.15 | 1.78 | 1.05 | 1.56 |
| Krt18 | 0.08 | 0.26 | 0.14 | 0.71 | 1.72 | 1.73 |
| Krt33a | 0.18 | 0.69 | 0.04 | 35.34 | 0.02 | 1.06 |
| Krt20 | 0.33 | 0.47 | 0.44 | 1.22 | 1.09 | 1.77 |
| Krt6a | 1450.28 | 183.01 | 496.41 | 2374.53 | 2456.42 | 2579.26 |
| Krt31 | 0.93 | 1.11 | 0.03 | 51.88 | 0.09 | 1.48 |
| Krt23 | 8.71 | 17.43 | 31.51 | 12.29 | 4.64 | 4.19 |
| Krt87 | 0.79 | 0.96 | 0.04 | 33.65 | 0.12 | 0.85 |
| Krt83 | 0.50 | 1.19 | 0.00 | 49.43 | 0.24 | 1.06 |
| Krt86 | 0.78 | 1.62 | 0.06 | 42.11 | 0.16 | 0.85 |
| Krt16 | 2233.91 | 723.08 | 1326.59 | 3119.17 | 2877.52 | 3232.26 |
| Krt17 | 828.84 | 504.45 | 382.56 | 1741.57 | 955.97 | 871.26 |
| Krtap11-1 | 0.71 | 2.25 | 0.05 | 73.35 | 0.09 | 1.03 |
| Krt90 | 0.23 | 0.02 | 0.04 | 0.87 | 0.23 | 0.44 |
| Krt6b | 1583.72 | 258.94 | 699.73 | 2312.77 | 2101.64 | 2206.26 |
| Krtap3-3 | 0.56 | 1.76 | 0.32 | 48.59 | 0.03 | 0.33 |
| Krtap8-1 | 1.36 | 2.14 | 0.00 | 149.49 | 0.00 | 1.27 |
| Krtap3-1 | 0.15 | 2.14 | 0.00 | 76.04 | 0.04 | 0.45 |
| Krtap7-1 | 1.87 | 5.51 | 0.00 | 187.09 | 0.07 | 2.97 |
| Krt26 | 0.23 | 0.30 | 0.01 | 7.94 | 0.01 | 0.05 |
| Krtap9-3 | 0.21 | 1.49 | 0.00 | 43.46 | 0.00 | 0.52 |
| Krt81 | 0.37 | 1.42 | 0.00 | 43.66 | 0.00 | 0.55 |
| Krt33b | 0.08 | 0.96 | 0.00 | 23.94 | 0.00 | 0.28 |
| Krtap16-1 | 0.11 | 0.48 | 0.00 | 11.12 | 0.00 | 0.13 |
| Krtap4-7 | 0.30 | 0.95 | 0.00 | 28.51 | 0.00 | 0.05 |
| Krtap9-1 | 0.11 | 0.95 | 0.00 | 18.31 | 0.00 | 0.04 |
| Krt72 | 0.72 | 2.40 | 0.00 | 41.08 | 0.02 | 0.32 |
| Krtap4-8 | 0.08 | 1.06 | 0.00 | 14.54 | 0.00 | 0.25 |
| Krtap1-3 | 0.24 | 1.41 | 0.00 | 21.13 | 0.00 | 0.28 |

Supplemental table 3. The FPKM values of important inflammatory genes.

| gene_name | FPKM.IMQ5_LTH_1 | FPKM.IMQ5_LTH_2 | FPKM.IMQ5_LTH_3 | FPKM.IMQ5_H2O_1 | FPKM.IMQ5_H2O_2 | FPKM.IMQ5_H2O_3 |
| --- | --- | --- | --- | --- | --- | --- |
| Il1f5 | 25.80 | 34.76 | 52.66 | 18.12 | 10.85 | 13.53 |
| Il18 | 15.03 | 28.56 | 45.96 | 10.50 | 10.74 | 9.52 |
| Il1b | 30.04 | 2.31 | 3.73 | 98.52 | 228.51 | 260.10 |
| Il33 | 25.55 | 3.67 | 6.26 | 69.03 | 42.73 | 54.49 |
| Il6 | 0.47 | 0.00 | 0.10 | 3.39 | 1.10 | 2.24 |
| Il23a | 0.25 | 0.12 | 0.00 | 0.92 | 0.58 | 0.72 |
| Cxcl2 | 7.19 | 0.87 | 1.51 | 67.28 | 150.76 | 143.49 |
| Cxcl13 | 91.98 | 112.30 | 62.04 | 232.36 | 316.88 | 524.67 |
| Cxcl3 | 1.16 | 0.02 | 0.09 | 10.13 | 22.81 | 19.60 |
| Cxcl5 | 3.61 | 0.02 | 0.08 | 43.34 | 16.98 | 17.68 |
| Cxcl1 | 3.46 | 0.07 | 0.07 | 23.29 | 8.68 | 11.17 |
| Ccl3 | 9.90 | 1.47 | 2.45 | 65.03 | 94.33 | 142.81 |
| Ccl4 | 6.04 | 0.96 | 1.85 | 35.74 | 57.43 | 138.26 |
| Ccl21b | 6.43 | 0.00 | 7.19 | 0.00 | 0.00 | 0.00 |
| Ccl24 | 1.29 | 1.24 | 2.72 | 0.29 | 0.33 | 0.32 |
| Ccl12 | 7.48 | 3.36 | 1.68 | 14.77 | 13.16 | 50.83 |
| Ccl6 | 194.76 | 50.98 | 85.05 | 266.66 | 425.37 | 451.92 |
| Ccl9 | 54.85 | 20.65 | 25.41 | 76.37 | 92.60 | 127.76 |
| Ccl7 | 49.11 | 9.00 | 9.67 | 47.36 | 30.76 | 74.16 |
| Tnf | 2.55 | 1.10 | 0.82 | 5.13 | 12.82 | 26.01 |
| S100a8 | 985.39 | 82.90 | 89.32 | 3174.95 | 2249.15 | 2502.52 |
| S100a9 | 3262.79 | 205.23 | 275.72 | 8607.27 | 6276.23 | 6533.72 |
| S100a7a | 3.55 | 0.44 | 0.15 | 10.75 | 7.81 | 7.33 |

Supplemental table 4. The FPKM values of significantly differentiated cell surface associated genes.

| gene_name | FPKM.IMQ5_LTH_1 | FPKM.IMQ5_LTH_2 | FPKM.IMQ5_LTH_3 | FPKM.IMQ5_H2O_1 | FPKM.IMQ5_H2O_2 | FPKM.IMQ5_H2O_3 |
| --- | --- | --- | --- | --- | --- | --- |
| Clstn3 | 6.24 | 8.46 | 7.17 | 1.16 | 0.56 | 0.83 |
| Car4 | 4.98 | 1.74 | 1.42 | 28.39 | 39.35 | 36.55 |
| Plaur | 5.92 | 2.73 | 2.83 | 18.19 | 47.09 | 43.61 |
| Cd14 | 23.42 | 12.89 | 8.44 | 74.06 | 168.98 | 184.19 |
| Wnt7b | 6.67 | 7.52 | 9.92 | 1.78 | 1.71 | 1.79 |
| Thbs1 | 7.79 | 4.43 | 3.47 | 22.81 | 28.68 | 29.16 |
| Sfrp4 | 7.70 | 4.92 | 8.02 | 1.17 | 1.17 | 1.70 |
| Il12a | 0.03 | 0.00 | 0.00 | 0.31 | 0.53 | 0.66 |
| Ntrk2 | 1.62 | 1.66 | 1.52 | 4.26 | 4.71 | 5.25 |
| Slc46a2 | 5.97 | 10.44 | 15.80 | 2.71 | 1.11 | 1.13 |
| Lrp4 | 4.57 | 9.28 | 8.96 | 2.75 | 2.13 | 1.68 |
| Cxcl13 | 91.98 | 112.30 | 62.04 | 232.36 | 316.88 | 524.67 |
| Cd38 | 5.90 | 2.59 | 2.16 | 12.00 | 14.85 | 26.66 |
| Cxcr2 | 4.28 | 1.09 | 1.37 | 9.72 | 18.02 | 16.03 |
| Tnf | 2.55 | 1.10 | 0.82 | 5.13 | 12.82 | 26.01 |
| Casr | 0.19 | 0.25 | 0.28 | 1.82 | 1.24 | 1.28 |
| Il1r1 | 7.78 | 6.28 | 5.01 | 23.48 | 17.19 | 15.40 |
| Fpr2 | 9.74 | 9.46 | 3.11 | 24.47 | 31.32 | 56.99 |
| Cd80 | 0.60 | 0.48 | 0.29 | 1.82 | 3.11 | 2.54 |
| Ighm | 41.85 | 53.44 | 43.84 | 12.06 | 15.40 | 23.44 |
| Treml4 | 0.25 | 0.09 | 0.22 | 0.90 | 1.87 | 3.69 |
| Fgfr2 | 5.72 | 8.78 | 6.57 | 3.32 | 2.76 | 2.83 |
| Slc7a11 | 1.94 | 0.63 | 0.81 | 5.73 | 3.95 | 3.66 |
| Cd53 | 29.78 | 21.20 | 16.74 | 47.66 | 64.87 | 103.25 |
| Tgfa | 0.42 | 1.97 | 2.20 | 9.28 | 7.94 | 6.96 |
| Tspear | 0.46 | 2.96 | 1.30 | 0.25 | 0.12 | 0.09 |
| Ccr5 | 2.10 | 1.67 | 0.91 | 5.04 | 3.68 | 5.59 |
| Ltf | 0.05 | 0.02 | 0.00 | 0.24 | 0.30 | 0.46 |
| Hhip | 0.23 | 0.34 | 0.14 | 0.05 | 0.04 | 0.02 |
| Ephb6 | 24.71 | 37.18 | 35.30 | 16.65 | 13.40 | 11.66 |
| Rtn4rl2 | 2.11 | 0.56 | 1.05 | 8.39 | 6.18 | 6.44 |
| Adamts15 | 3.98 | 3.28 | 1.94 | 6.36 | 8.80 | 9.81 |
| Ndp | 1.32 | 1.61 | 0.59 | 0.17 | 0.12 | 0.25 |
| P2rx2 | 0.23 | 0.71 | 0.39 | 0.05 | 0.08 | 0.04 |
| Areg | 6.55 | 1.16 | 1.36 | 20.40 | 16.37 | 21.89 |
| Ramp1 | 6.21 | 11.86 | 8.32 | 3.72 | 3.12 | 2.77 |
| Anxa9 | 12.68 | 22.86 | 34.04 | 10.13 | 6.87 | 6.89 |
| Ptprc | 1.88 | 1.29 | 0.91 | 2.77 | 3.39 | 5.16 |
| Clcf1 | 6.52 | 2.96 | 1.30 | 11.01 | 16.31 | 16.06 |
| A530064D06Rik | 0.18 | 0.12 | 0.06 | 0.46 | 0.80 | 0.76 |
| Prlr | 0.39 | 0.66 | 0.57 | 0.12 | 0.12 | 0.20 |
| Fcgr4 | 16.67 | 23.39 | 8.36 | 38.28 | 38.30 | 93.62 |
| Rtn4r | 3.24 | 5.43 | 3.65 | 1.14 | 1.12 | 1.34 |
| Treml2 | 0.03 | 0.00 | 0.04 | 0.16 | 0.54 | 0.19 |
| Adam8 | 11.67 | 4.88 | 7.68 | 16.67 | 26.18 | 26.23 |
| Fgf7 | 2.41 | 1.83 | 1.35 | 5.62 | 5.38 | 5.48 |
| Vcam1 | 0.81 | 0.70 | 0.59 | 1.59 | 2.51 | 2.03 |
| Sell | 1.20 | 0.15 | 0.23 | 2.16 | 4.17 | 4.40 |
| Hbegf | 5.42 | 1.86 | 1.79 | 13.37 | 8.11 | 8.57 |
| Adam3 | 0.68 | 1.02 | 0.99 | 0.31 | 0.12 | 0.15 |
| Adgrf4 | 17.14 | 17.45 | 22.52 | 11.36 | 8.19 | 8.73 |
| Lipg | 2.30 | 1.08 | 0.83 | 4.06 | 3.52 | 8.39 |
| Fcgr1 | 7.52 | 8.35 | 4.54 | 15.53 | 11.89 | 29.96 |
| Scube3 | 2.30 | 1.04 | 0.98 | 0.59 | 0.23 | 0.31 |
| Mrc1 | 25.18 | 14.41 | 18.17 | 37.01 | 47.13 | 49.63 |
| Ambp | 0.37 | 0.13 | 0.23 | 1.12 | 0.89 | 1.84 |
| Nrros | 8.95 | 8.32 | 8.80 | 14.56 | 17.67 | 21.45 |
| Cd177 | 0.91 | 0.02 | 0.07 | 2.36 | 6.49 | 6.88 |
| Adgrg3 | 0.67 | 0.55 | 0.44 | 1.17 | 1.92 | 2.13 |
| Folh1 | 0.04 | 0.10 | 0.10 | 0.24 | 0.49 | 0.49 |
| Entpd1 | 9.31 | 6.11 | 5.76 | 13.71 | 21.18 | 12.91 |
| Amot | 4.54 | 6.12 | 6.17 | 1.45 | 3.69 | 2.18 |
| Edn3 | 0.64 | 0.96 | 0.17 | 0.07 | 0.17 | 0.08 |
| Enpp1 | 0.66 | 0.30 | 0.33 | 1.39 | 1.16 | 1.48 |
| Ncl | 23.03 | 20.65 | 17.77 | 46.78 | 42.09 | 36.38 |
| Plscr1 | 2.64 | 1.94 | 1.42 | 5.40 | 4.83 | 6.80 |
| Clec5a | 0.72 | 0.40 | 0.42 | 1.40 | 1.67 | 2.17 |
| Wnt6 | 2.74 | 3.82 | 2.46 | 1.26 | 0.95 | 0.86 |
| Ptn | 5.32 | 12.21 | 6.19 | 4.45 | 2.20 | 1.75 |
| Picalm | 7.79 | 6.37 | 5.34 | 12.19 | 13.28 | 13.99 |
| Anxa1 | 99.02 | 61.15 | 76.13 | 176.09 | 197.57 | 138.82 |
| Tmprss11d | 0.26 | 0.39 | 1.01 | 0.13 | 0.13 | 0.08 |
| Tnfrsf22 | 1.11 | 0.87 | 0.73 | 2.28 | 2.75 | 2.47 |
| Cdh4 | 0.35 | 0.69 | 0.69 | 0.16 | 0.23 | 0.17 |
| Ctss | 93.05 | 81.51 | 72.44 | 136.47 | 157.06 | 216.31 |
| Msn | 42.84 | 27.20 | 22.36 | 59.84 | 61.94 | 75.86 |
| Itgb2 | 31.05 | 26.59 | 14.55 | 44.69 | 41.06 | 96.70 |
| Lyn | 12.99 | 9.51 | 5.24 | 17.76 | 16.20 | 35.00 |
| Tlr4 | 0.16 | 1.27 | 0.40 | 1.92 | 2.87 | 3.09 |
| Lrrc32 | 11.06 | 7.21 | 4.87 | 16.48 | 15.11 | 16.02 |
| Tnfrsf26 | 0.83 | 1.32 | 0.78 | 2.21 | 2.58 | 3.54 |
| Tyrobp | 172.94 | 124.27 | 112.52 | 215.81 | 255.43 | 414.35 |
| Cyp2w1 | 0.18 | 0.59 | 1.25 | 0.08 | 0.21 | 0.04 |
| Nod2 | 3.56 | 1.64 | 1.30 | 7.69 | 4.75 | 4.69 |
| Bmp2 | 1.39 | 3.15 | 2.59 | 1.10 | 0.62 | 0.85 |
| Thbd | 18.42 | 10.19 | 11.61 | 29.46 | 28.02 | 23.56 |
| Slc28a2 | 3.43 | 2.86 | 1.80 | 4.99 | 4.92 | 9.77 |
| Duox1 | 4.90 | 2.04 | 2.28 | 8.48 | 6.78 | 6.14 |
| Ace2 | 9.87 | 9.36 | 12.91 | 7.54 | 5.05 | 2.72 |
| Scnn1b | 5.88 | 5.29 | 5.23 | 2.64 | 2.75 | 2.55 |
| Syk | 7.16 | 4.69 | 4.03 | 7.62 | 11.10 | 15.00 |
| Epcam | 20.92 | 26.72 | 23.65 | 18.67 | 8.01 | 8.16 |
| Kcnc1 | 2.30 | 2.71 | 2.41 | 0.69 | 1.69 | 1.10 |
| Adgrb2 | 0.64 | 0.46 | 0.64 | 0.25 | 0.21 | 0.21 |
| Acvrl1 | 4.25 | 4.46 | 3.25 | 10.73 | 6.46 | 7.08 |
| Il1rl1 | 0.48 | 0.35 | 0.49 | 0.88 | 1.20 | 1.48 |
| Celsr3 | 0.13 | 0.13 | 0.14 | 0.32 | 0.42 | 0.37 |
| Tlr2 | 6.56 | 3.54 | 3.25 | 6.11 | 10.55 | 17.19 |
| Scube1 | 0.55 | 0.71 | 0.38 | 0.29 | 0.15 | 0.05 |
| Cdon | 4.40 | 4.05 | 3.69 | 1.52 | 3.13 | 1.39 |
| Corin | 0.63 | 1.18 | 0.35 | 0.33 | 0.11 | 0.03 |
| Tnfrsf1b | 16.70 | 13.59 | 9.77 | 20.06 | 22.05 | 38.78 |
| Tfpi | 1.30 | 1.99 | 1.64 | 3.22 | 2.86 | 4.39 |
| Cxcr1 | 0.30 | 0.00 | 0.00 | 0.66 | 1.00 | 1.73 |
| Slc1a3 | 1.90 | 0.84 | 1.44 | 3.09 | 3.34 | 2.62 |
| Hspa5 | 60.90 | 42.27 | 14.81 | 80.34 | 87.12 | 97.21 |
| Wnt3a | 1.54 | 1.16 | 1.35 | 2.74 | 3.31 | 3.23 |
| Adgre4 | 0.60 | 0.35 | 0.37 | 0.80 | 1.18 | 1.57 |
| B430306N03Rik | 0.57 | 0.12 | 0.06 | 1.00 | 0.76 | 1.26 |
| Lrp8 | 0.34 | 0.23 | 0.09 | 0.54 | 0.81 | 0.47 |
| 9830107B12Rik | 0.10 | 0.00 | 0.00 | 0.14 | 0.30 | 0.54 |
| Mlkl | 4.37 | 3.08 | 3.55 | 6.92 | 7.05 | 8.18 |
| Nfam1 | 7.61 | 2.25 | 4.11 | 7.58 | 9.71 | 13.34 |
| Kcnh2 | 0.33 | 0.57 | 0.58 | 0.15 | 0.26 | 0.18 |
| Tacr1 | 0.69 | 0.96 | 0.67 | 1.49 | 1.79 | 1.73 |
| Mmp25 | 0.35 | 0.09 | 0.10 | 0.52 | 0.59 | 0.58 |
| Heg1 | 1.69 | 0.44 | 0.54 | 2.34 | 1.89 | 2.18 |
| Grem1 | 1.37 | 1.76 | 1.97 | 2.36 | 3.88 | 4.66 |
| Cntn2 | 0.08 | 0.03 | 0.01 | 0.33 | 0.05 | 0.17 |
| Ramp3 | 3.16 | 3.55 | 3.47 | 1.35 | 1.66 | 1.91 |
| Cxcr4 | 5.01 | 5.92 | 2.62 | 5.91 | 10.58 | 11.95 |
| Tmem8b | 2.29 | 3.53 | 2.68 | 1.35 | 2.15 | 0.51 |
| Ceacam1 | 2.37 | 1.79 | 0.55 | 2.75 | 3.81 | 3.75 |
| Cspg5 | 0.12 | 0.09 | 0.08 | 0.05 | 0.02 | 0.04 |
| Cacna1d | 0.39 | 0.21 | 0.44 | 0.42 | 0.74 | 1.31 |
| Cd69 | 0.10 | 0.12 | 0.07 | 0.18 | 0.17 | 0.41 |
| Scube2 | 0.33 | 0.44 | 0.56 | 0.22 | 0.22 | 0.16 |
| Crhr1 | 0.57 | 0.88 | 0.18 | 0.22 | 0.00 | 0.00 |
| Fcer1a | 2.06 | 1.85 | 1.08 | 3.54 | 3.24 | 3.86 |
| Mrgprb2 | 0.42 | 1.20 | 1.27 | 0.44 | 0.36 | 0.47 |
| Fcrl5 | 0.60 | 1.05 | 0.11 | 1.05 | 0.61 | 4.33 |
| Col4a3 | 0.18 | 0.29 | 0.24 | 0.16 | 0.07 | 0.10 |
| Shh | 2.13 | 3.27 | 0.68 | 1.08 | 0.02 | 0.03 |
| Itgav | 0.29 | 0.14 | 0.19 | 0.46 | 0.39 | 0.46 |
| Adam10 | 10.70 | 2.08 | 6.71 | 13.54 | 11.96 | 13.68 |
| Fut4 | 0.37 | 0.26 | 0.19 | 0.55 | 0.43 | 0.76 |
| Ccr10 | 0.16 | 0.54 | 0.69 | 0.12 | 0.20 | 0.27 |
| Hcst | 2.29 | 2.01 | 2.21 | 2.84 | 5.21 | 5.14 |
| Ostn | 0.38 | 0.69 | 0.88 | 0.00 | 0.32 | 0.32 |

Supplemental table 5. The FPKM values of significantly differentiated extracellular region associated genes.

| gene_name | FPKM.IMQ5_LTH_1 | FPKM.IMQ5_LTH_2 | FPKM.IMQ5_LTH_3 | FPKM.IMQ5_H2O_1 | FPKM.IMQ5_H2O_2 | FPKM.IMQ5_H2O_3 |
| --- | --- | --- | --- | --- | --- | --- |
| Prl2c3 | 0.00 | 0.00 | 0.00 | 2.43 | 4.86 | 5.10 |
| Serpina3b | 20.82 | 104.32 | 76.08 | 0.97 | 0.39 | 0.32 |
| 4930486L24Rik | 0.00 | 0.00 | 0.00 | 0.58 | 1.30 | 1.60 |
| Gm7298 | 0.91 | 2.68 | 1.83 | 0.00 | 0.00 | 0.03 |
| Saa1 | 6.94 | 2.36 | 2.41 | 33.13 | 34.44 | 41.16 |
| Plaur | 5.92 | 2.73 | 2.83 | 18.19 | 47.09 | 43.61 |
| Ngp | 0.24 | 0.00 | 0.08 | 8.45 | 6.49 | 11.54 |
| Srgn | 22.81 | 10.07 | 9.85 | 78.98 | 122.41 | 161.41 |
| Spp1 | 5.52 | 2.58 | 2.59 | 17.51 | 59.47 | 75.22 |
| Cxcl2 | 7.19 | 0.87 | 1.51 | 67.28 | 150.76 | 143.49 |
| Cd14 | 23.42 | 12.89 | 8.44 | 74.06 | 168.98 | 184.19 |
| Gc | 0.00 | 0.00 | 0.00 | 0.27 | 0.48 | 1.38 |
| Wnt7b | 6.67 | 7.52 | 9.92 | 1.78 | 1.71 | 1.79 |
| Prl2c2 | 0.00 | 0.00 | 0.00 | 0.29 | 1.01 | 0.93 |
| Ccl3 | 9.90 | 1.47 | 2.45 | 65.03 | 94.33 | 142.81 |
| Thbs1 | 7.79 | 4.43 | 3.47 | 22.81 | 28.68 | 29.16 |
| Chit1 | 65.45 | 82.79 | 125.85 | 24.00 | 14.47 | 13.94 |
| Ces4a | 31.98 | 19.13 | 18.60 | 6.20 | 3.31 | 2.40 |
| Ccl4 | 6.04 | 0.96 | 1.85 | 35.74 | 57.43 | 138.26 |
| Sfrp4 | 7.70 | 4.92 | 8.02 | 1.17 | 1.17 | 1.70 |
| Pla2g7 | 14.76 | 11.04 | 10.66 | 38.89 | 42.28 | 56.09 |
| Retnlg | 10.36 | 0.58 | 0.94 | 203.19 | 216.88 | 347.50 |
| Chil3 | 14.46 | 2.44 | 4.32 | 58.38 | 105.21 | 121.41 |
| Saa2 | 6.81 | 1.97 | 2.63 | 21.83 | 29.72 | 29.01 |
| Il12a | 0.03 | 0.00 | 0.00 | 0.31 | 0.53 | 0.66 |
| Nrtn | 19.54 | 43.35 | 43.61 | 8.01 | 5.98 | 6.00 |
| Retnla | 24.93 | 30.77 | 66.76 | 2.49 | 3.39 | 6.98 |
| Gm94 | 77.35 | 116.10 | 243.91 | 33.18 | 17.74 | 16.26 |
| Serpine1 | 1.57 | 1.33 | 1.12 | 6.67 | 4.66 | 8.91 |
| Slurp2 | 5.69 | 26.11 | 24.20 | 1.59 | 0.42 | 0.36 |
| Mmp8 | 1.94 | 0.13 | 0.37 | 12.99 | 18.53 | 30.78 |
| Orm1 | 61.13 | 74.74 | 95.08 | 29.66 | 21.70 | 23.96 |
| Pla2g2f | 95.08 | 117.22 | 185.43 | 44.04 | 32.44 | 35.19 |
| Slurp1 | 1.75 | 6.73 | 7.29 | 0.59 | 0.18 | 0.10 |
| Cp | 3.93 | 3.29 | 2.27 | 9.23 | 9.35 | 10.18 |
| Hmcn2 | 3.83 | 4.94 | 4.22 | 1.73 | 1.67 | 0.97 |
| Prok2 | 0.12 | 0.05 | 0.00 | 1.50 | 1.22 | 1.65 |
| C1qtnf3 | 3.46 | 8.06 | 3.61 | 1.00 | 0.80 | 0.59 |
| Cxcl13 | 91.98 | 112.30 | 62.04 | 232.36 | 316.88 | 524.67 |
| Serpina12 | 3.25 | 9.81 | 20.97 | 1.65 | 0.39 | 0.64 |
| Serpina3m | 4.68 | 2.53 | 2.21 | 11.13 | 17.21 | 15.52 |
| Pglyrp1 | 3.31 | 1.05 | 1.48 | 9.40 | 11.82 | 14.09 |
| Defb14 | 43.59 | 18.60 | 33.69 | 165.67 | 133.87 | 122.93 |
| Tnf | 2.55 | 1.10 | 0.82 | 5.13 | 12.82 | 26.01 |
| Il1r1 | 7.78 | 6.28 | 5.01 | 23.48 | 17.19 | 15.40 |
| Slpi | 28.41 | 3.94 | 5.57 | 91.29 | 114.24 | 156.49 |
| Ccl21b | 6.43 | 0.00 | 7.19 | 0.00 | 0.00 | 0.00 |
| Il4ra | 27.71 | 11.36 | 11.57 | 66.93 | 60.26 | 65.24 |
| Fgf23 | 0.05 | 0.00 | 0.00 | 4.46 | 0.44 | 0.55 |
| Nmu | 0.42 | 2.20 | 2.13 | 0.11 | 0.00 | 0.00 |
| Cxcl3 | 1.16 | 0.02 | 0.09 | 10.13 | 22.81 | 19.60 |
| Mmp9 | 5.05 | 0.85 | 2.26 | 10.83 | 19.35 | 25.03 |
| Angptl4 | 9.57 | 17.75 | 5.33 | 47.95 | 42.36 | 32.89 |
| Nlrp3 | 1.11 | 0.30 | 0.31 | 1.49 | 11.46 | 8.22 |
| Il1b | 30.04 | 2.31 | 3.73 | 98.52 | 228.51 | 260.10 |
| Angptl8 | 3.51 | 18.04 | 7.51 | 0.28 | 0.99 | 1.36 |
| Wfdc17 | 223.64 | 127.88 | 113.53 | 375.67 | 491.58 | 715.78 |
| Dkkl1 | 11.56 | 14.08 | 39.51 | 5.50 | 3.92 | 4.31 |
| Klk5 | 45.89 | 72.86 | 121.23 | 27.56 | 20.51 | 27.08 |
| Reg3g | 0.07 | 0.02 | 0.00 | 0.24 | 0.67 | 1.00 |
| Cdcp3 | 0.17 | 0.26 | 0.33 | 0.06 | 0.03 | 0.03 |
| Myoc | 6.67 | 14.44 | 17.81 | 4.25 | 2.58 | 1.39 |
| Wnt3 | 3.53 | 4.21 | 6.59 | 1.48 | 0.71 | 1.17 |
| Tgfa | 0.42 | 1.97 | 2.20 | 9.28 | 7.94 | 6.96 |
| Chga | 0.79 | 2.05 | 3.65 | 0.30 | 0.17 | 0.36 |
| Tspear | 0.46 | 2.96 | 1.30 | 0.25 | 0.12 | 0.09 |
| Ctla2a | 9.77 | 6.28 | 6.49 | 14.92 | 24.98 | 26.19 |
| Cemip | 0.52 | 0.23 | 0.45 | 1.08 | 1.96 | 2.45 |
| Ptprz1 | 0.70 | 0.39 | 0.51 | 1.83 | 2.09 | 1.44 |
| Reg1 | 0.30 | 0.05 | 0.00 | 1.45 | 2.63 | 2.63 |
| Osm | 1.61 | 0.27 | 0.30 | 3.47 | 8.79 | 9.34 |
| Tnfrsf19 | 7.08 | 10.84 | 6.83 | 4.11 | 1.83 | 1.67 |
| Csf3 | 0.77 | 0.00 | 0.17 | 10.45 | 6.02 | 10.90 |
| Il20 | 1.17 | 4.00 | 1.13 | 0.36 | 0.08 | 0.11 |
| Saa3 | 289.76 | 62.36 | 29.16 | 843.75 | 810.95 | 1468.77 |
| Ltf | 0.05 | 0.02 | 0.00 | 0.24 | 0.30 | 0.46 |
| Hhip | 0.23 | 0.34 | 0.14 | 0.05 | 0.04 | 0.02 |
| Prxl2a | 17.56 | 29.10 | 28.24 | 10.26 | 11.49 | 11.05 |
| Ephb6 | 24.71 | 37.18 | 35.30 | 16.65 | 13.40 | 11.66 |
| Wfdc12 | 11.54 | 41.20 | 77.81 | 6.05 | 4.50 | 10.82 |
| F10 | 1.29 | 0.25 | 0.19 | 3.48 | 4.91 | 7.03 |
| Adamts15 | 3.98 | 3.28 | 1.94 | 6.36 | 8.80 | 9.81 |
| Ndp | 1.32 | 1.61 | 0.59 | 0.17 | 0.12 | 0.25 |
| Il1r2 | 14.20 | 20.91 | 22.78 | 35.57 | 66.34 | 65.42 |
| Tuft1 | 18.08 | 26.58 | 33.24 | 12.37 | 10.32 | 10.42 |
| Pon1 | 0.09 | 1.17 | 0.84 | 0.05 | 0.02 | 0.07 |
| Defb6 | 567.94 | 537.49 | 529.99 | 318.31 | 147.68 | 169.24 |
| Ccl24 | 1.29 | 1.24 | 2.72 | 0.29 | 0.33 | 0.32 |
| Il1f5 | 25.80 | 34.76 | 52.66 | 18.12 | 10.85 | 13.53 |
| Hpx | 5.15 | 1.40 | 0.94 | 13.01 | 10.54 | 23.05 |
| Il1rap | 1.25 | 0.94 | 1.10 | 2.39 | 3.10 | 3.41 |
| Calcb | 2.81 | 2.83 | 3.17 | 0.50 | 0.39 | 0.93 |
| Rptn | 68.06 | 90.37 | 126.79 | 50.58 | 23.69 | 26.10 |
| Hp | 879.85 | 304.34 | 351.98 | 1743.16 | 1499.99 | 1760.64 |
| Clcf1 | 6.52 | 2.96 | 1.30 | 11.01 | 16.31 | 16.06 |
| Lamc3 | 1.87 | 3.67 | 2.06 | 1.01 | 0.59 | 0.70 |
| Ptx3 | 3.49 | 0.72 | 0.79 | 9.39 | 6.94 | 11.27 |
| Acpp | 13.39 | 14.96 | 24.93 | 9.61 | 4.71 | 4.66 |
| Igfbp6 | 54.01 | 100.73 | 94.79 | 33.92 | 34.88 | 40.58 |
| Vwa2 | 4.20 | 7.50 | 5.05 | 2.20 | 2.17 | 2.09 |
| Mst1 | 3.70 | 6.19 | 6.53 | 1.69 | 1.78 | 1.67 |
| Pcolce2 | 6.60 | 10.04 | 10.14 | 2.58 | 4.27 | 4.42 |
| Nrg4 | 2.00 | 2.56 | 2.86 | 0.86 | 0.99 | 0.41 |
| Lrrc17 | 7.40 | 7.11 | 7.01 | 2.92 | 3.40 | 3.10 |
| Guca2a | 1.32 | 3.08 | 4.38 | 0.00 | 0.08 | 0.48 |
| Fgf7 | 2.41 | 1.83 | 1.35 | 5.62 | 5.38 | 5.48 |
| Cpa4 | 22.97 | 32.03 | 65.79 | 16.82 | 14.67 | 12.57 |
| Klk11 | 44.66 | 54.20 | 63.19 | 32.48 | 23.37 | 21.40 |
| Apol7a | 0.30 | 0.32 | 0.65 | 0.09 | 0.08 | 0.07 |
| Cxcl12 | 13.89 | 7.99 | 6.85 | 22.57 | 22.32 | 25.92 |
| Lcn2 | 94.10 | 9.78 | 11.72 | 311.05 | 239.95 | 248.74 |
| Spink7 | 15.60 | 18.81 | 60.98 | 4.16 | 7.52 | 9.38 |
| Hbegf | 5.42 | 1.86 | 1.79 | 13.37 | 8.11 | 8.57 |
| Lipg | 2.30 | 1.08 | 0.83 | 4.06 | 3.52 | 8.39 |
| Il18 | 15.03 | 28.56 | 45.96 | 10.50 | 10.74 | 9.52 |
| Ccl12 | 7.48 | 3.36 | 1.68 | 14.77 | 13.16 | 50.83 |
| Lama3 | 3.28 | 3.22 | 2.94 | 5.67 | 7.35 | 6.42 |
| Ccl6 | 194.76 | 50.98 | 85.05 | 266.66 | 425.37 | 451.92 |
| Ccl9 | 54.85 | 20.65 | 25.41 | 76.37 | 92.60 | 127.76 |
| Olr1 | 0.12 | 0.05 | 0.05 | 0.32 | 0.39 | 0.48 |
| Tmprss2 | 0.15 | 0.08 | 0.06 | 0.47 | 0.45 | 0.38 |
| Wfdc3 | 1.56 | 2.63 | 2.59 | 0.54 | 0.09 | 0.59 |
| Dnase1l2 | 24.17 | 25.42 | 39.87 | 15.80 | 11.38 | 14.90 |
| Scube3 | 2.30 | 1.04 | 0.98 | 0.59 | 0.23 | 0.31 |
| Il33 | 25.55 | 3.67 | 6.26 | 69.03 | 42.73 | 54.49 |
| Egfl6 | 0.67 | 2.10 | 0.99 | 0.38 | 0.24 | 0.14 |
| Dmkn | 567.94 | 539.72 | 1018.24 | 337.59 | 298.32 | 320.94 |
| Cdsn | 100.30 | 119.96 | 237.60 | 63.76 | 62.80 | 64.50 |
| Mstn | 1.53 | 4.94 | 1.75 | 0.94 | 0.53 | 0.34 |
| Tmprss11f | 0.49 | 0.57 | 1.20 | 0.25 | 0.20 | 0.11 |
| Fgf6 | 0.30 | 0.27 | 0.25 | 0.05 | 0.09 | 0.01 |
| Ambp | 0.37 | 0.13 | 0.23 | 1.12 | 0.89 | 1.84 |
| Nrros | 8.95 | 8.32 | 8.80 | 14.56 | 17.67 | 21.45 |
| Tnc | 58.25 | 9.45 | 11.44 | 104.48 | 123.91 | 122.07 |
| Rarres2 | 99.69 | 45.49 | 55.64 | 157.82 | 161.96 | 169.40 |
| Crispld2 | 9.90 | 5.47 | 6.72 | 13.44 | 17.31 | 19.29 |
| Cfb | 36.21 | 27.05 | 18.30 | 64.56 | 50.84 | 69.03 |
| Olfm2 | 2.47 | 2.88 | 3.50 | 0.96 | 1.25 | 0.85 |
| Cd163 | 23.50 | 12.34 | 17.71 | 31.21 | 45.73 | 55.19 |
| Edn3 | 0.64 | 0.96 | 0.17 | 0.07 | 0.17 | 0.08 |
| Angptl1 | 2.09 | 2.63 | 2.36 | 0.93 | 0.74 | 0.85 |
| S100a8 | 985.39 | 82.90 | 89.32 | 3174.95 | 2249.15 | 2502.52 |
| Wnt6 | 2.74 | 3.82 | 2.46 | 1.26 | 0.95 | 0.86 |
| Pamr1 | 1.14 | 3.06 | 3.67 | 0.43 | 0.84 | 1.05 |
| Ptch1 | 3.05 | 3.92 | 1.56 | 1.68 | 1.01 | 0.80 |
| Draxin | 1.84 | 1.22 | 0.45 | 3.66 | 3.49 | 4.40 |
| Wnt2 | 0.47 | 1.07 | 0.80 | 0.28 | 0.15 | 0.13 |
| Lgals3bp | 14.66 | 16.28 | 13.60 | 34.42 | 32.93 | 22.48 |
| Ptn | 5.32 | 12.21 | 6.19 | 4.45 | 2.20 | 1.75 |
| Klk14 | 10.56 | 2.11 | 2.29 | 29.82 | 17.93 | 15.83 |
| Wfdc5 | 9.15 | 15.28 | 22.85 | 8.92 | 5.66 | 4.82 |
| Anxa1 | 99.02 | 61.15 | 76.13 | 176.09 | 197.57 | 138.82 |
| Defb3 | 40.09 | 2.02 | 3.49 | 154.06 | 108.06 | 103.53 |
| Col6a6 | 0.63 | 1.11 | 0.71 | 0.20 | 0.24 | 0.38 |
| Sema3e | 0.26 | 0.65 | 0.52 | 0.20 | 0.10 | 0.08 |
| Frem1 | 0.46 | 0.96 | 0.39 | 0.22 | 0.13 | 0.22 |
| Tmprss11d | 0.26 | 0.39 | 1.01 | 0.13 | 0.13 | 0.08 |
| Cxcl5 | 3.61 | 0.02 | 0.08 | 43.34 | 16.98 | 17.68 |
| Wnt10b | 2.00 | 3.04 | 0.82 | 0.79 | 0.16 | 0.24 |
| Tnfrsf22 | 1.11 | 0.87 | 0.73 | 2.28 | 2.75 | 2.47 |
| Gstm1 | 53.13 | 95.66 | 95.19 | 28.11 | 44.83 | 46.07 |
| Prelp | 24.51 | 18.99 | 13.78 | 38.36 | 34.86 | 43.07 |
| Pcsk5 | 2.13 | 1.13 | 0.71 | 3.58 | 3.30 | 2.79 |
| Kazald1 | 3.08 | 5.67 | 3.94 | 1.23 | 1.74 | 1.66 |
| Mmp10 | 0.44 | 0.09 | 0.14 | 0.40 | 2.26 | 1.57 |
| Pi15 | 5.99 | 1.51 | 1.34 | 11.06 | 8.69 | 8.22 |
| Tmprss11g | 0.63 | 0.39 | 0.29 | 3.50 | 1.31 | 0.70 |
| Serpina1e | 0.00 | 0.22 | 0.14 | 0.92 | 0.26 | 4.56 |
| Il6 | 0.47 | 0.00 | 0.10 | 3.39 | 1.10 | 2.24 |
| Tmem25 | 0.57 | 1.25 | 0.74 | 0.17 | 0.35 | 0.25 |
| Angpt2 | 1.86 | 1.87 | 1.40 | 3.70 | 4.12 | 4.23 |
| Olfm4 | 0.12 | 0.00 | 0.00 | 0.35 | 0.67 | 0.90 |
| Bmp2 | 1.39 | 3.15 | 2.59 | 1.10 | 0.62 | 0.85 |
| Apol8 | 2.85 | 2.75 | 3.53 | 1.42 | 1.24 | 1.17 |
| Rbp4 | 9.91 | 14.93 | 11.78 | 5.70 | 2.96 | 6.68 |
| Mettl24 | 0.93 | 2.34 | 1.92 | 0.32 | 0.37 | 0.76 |
| Defb1 | 56.06 | 22.79 | 36.32 | 101.45 | 85.46 | 67.73 |
| Ace2 | 9.87 | 9.36 | 12.91 | 7.54 | 5.05 | 2.72 |
| Aqp4 | 0.48 | 1.48 | 1.23 | 0.37 | 0.43 | 0.21 |
| Ccdc3 | 5.56 | 8.28 | 7.79 | 3.54 | 3.85 | 3.35 |
| Col4a4 | 0.60 | 0.92 | 0.76 | 0.26 | 0.39 | 0.27 |
| Klk6 | 150.60 | 52.75 | 111.11 | 278.19 | 213.63 | 248.70 |
| Cxcl1 | 3.46 | 0.07 | 0.07 | 23.29 | 8.68 | 11.17 |
| S100a9 | 3262.79 | 205.23 | 275.72 | 8607.27 | 6276.23 | 6533.72 |
| Adamts5 | 4.69 | 2.40 | 2.44 | 6.13 | 6.10 | 7.02 |
| Adamts14 | 2.06 | 5.96 | 4.97 | 1.67 | 1.56 | 2.27 |
| Tnfsf14 | 1.76 | 0.11 | 0.37 | 3.94 | 4.44 | 3.88 |
| Lipm | 9.57 | 19.04 | 24.13 | 11.13 | 7.99 | 6.96 |
| Sfrp2 | 5.07 | 5.72 | 3.50 | 8.87 | 9.01 | 13.36 |
| Il1rl1 | 0.48 | 0.35 | 0.49 | 0.88 | 1.20 | 1.48 |
| Gkn3 | 0.90 | 2.40 | 1.62 | 0.11 | 0.52 | 0.55 |
| Itih4 | 0.04 | 0.06 | 0.16 | 0.34 | 0.28 | 0.47 |
| Emilin2 | 13.12 | 9.10 | 8.97 | 15.14 | 18.77 | 29.18 |
| Bmper | 0.97 | 0.34 | 0.59 | 2.62 | 1.67 | 1.46 |
| Dhrs7c | 6.51 | 13.40 | 10.97 | 5.81 | 5.07 | 2.56 |
| Scube1 | 0.55 | 0.71 | 0.38 | 0.29 | 0.15 | 0.05 |
| Lgmn | 177.14 | 116.16 | 101.34 | 209.68 | 242.35 | 342.78 |
| Defb4 | 7.46 | 0.31 | 0.49 | 23.16 | 23.65 | 12.60 |
| Corin | 0.63 | 1.18 | 0.35 | 0.33 | 0.11 | 0.03 |
| Apoc2 | 3.90 | 1.47 | 2.73 | 5.70 | 6.69 | 13.36 |
| Fibin | 2.31 | 1.40 | 1.28 | 3.55 | 3.67 | 5.87 |
| Timp4 | 1.88 | 2.54 | 2.14 | 2.88 | 6.11 | 5.07 |
| Col27a1 | 1.25 | 1.65 | 0.74 | 0.73 | 0.48 | 0.44 |
| a | 1.13 | 1.42 | 1.16 | 2.84 | 3.50 | 3.38 |
| Tmprss11b | 0.46 | 0.14 | 0.03 | 1.19 | 1.09 | 0.66 |
| Gdnf | 0.36 | 0.51 | 0.35 | 0.76 | 1.22 | 1.37 |
| Fgl1 | 0.16 | 0.06 | 0.02 | 0.18 | 0.60 | 0.36 |
| Angptl7 | 0.33 | 1.33 | 2.71 | 0.41 | 0.25 | 0.45 |
| Agrp | 1.02 | 1.40 | 2.48 | 0.63 | 0.60 | 0.35 |
| Stc2 | 3.68 | 1.97 | 2.18 | 5.63 | 5.80 | 4.69 |
| Ngf | 2.40 | 2.02 | 2.33 | 5.65 | 4.54 | 6.11 |
| Tfpi | 1.30 | 1.99 | 1.64 | 3.22 | 2.86 | 4.39 |
| 1700029I15Rik | 2.65 | 5.15 | 3.35 | 1.75 | 1.40 | 1.46 |
| Il23a | 0.25 | 0.12 | 0.00 | 0.92 | 0.58 | 0.72 |
| Hspa5 | 60.90 | 42.27 | 14.81 | 80.34 | 87.12 | 97.21 |
| Notum | 0.84 | 2.69 | 2.40 | 1.09 | 0.15 | 0.17 |
| F5 | 0.18 | 0.11 | 0.04 | 0.26 | 0.45 | 0.37 |
| Nts | 0.46 | 0.25 | 0.15 | 0.95 | 0.70 | 1.17 |
| Mmp13 | 2.63 | 0.14 | 0.90 | 3.76 | 7.14 | 5.22 |
| Ppbp | 0.53 | 0.13 | 0.53 | 0.88 | 1.89 | 1.71 |
| Napsa | 4.56 | 1.82 | 2.03 | 4.47 | 6.56 | 10.10 |
| Col8a2 | 0.48 | 1.39 | 1.05 | 0.54 | 0.08 | 0.23 |
| Wnt3a | 1.54 | 1.16 | 1.35 | 2.74 | 3.31 | 3.23 |
| Svep1 | 5.54 | 2.18 | 2.00 | 5.52 | 6.99 | 7.96 |
| Fam180a | 1.59 | 3.15 | 3.31 | 0.74 | 0.71 | 1.60 |
| Lrp8 | 0.34 | 0.23 | 0.09 | 0.54 | 0.81 | 0.47 |
| Il22ra2 | 1.14 | 1.24 | 1.55 | 0.39 | 0.81 | 0.23 |
| Gm1673 | 1.52 | 2.55 | 2.39 | 0.69 | 0.62 | 1.09 |
| Glt1d1 | 1.81 | 0.91 | 0.86 | 3.37 | 2.49 | 2.55 |
| Ecm2 | 1.62 | 3.65 | 2.70 | 1.49 | 1.37 | 1.12 |
| Pla1a | 4.68 | 2.49 | 3.39 | 4.35 | 7.23 | 15.92 |
| Cbln1 | 1.81 | 0.34 | 0.26 | 1.33 | 4.32 | 3.64 |
| Glipr1 | 2.29 | 1.45 | 1.81 | 2.52 | 4.55 | 7.70 |
| Vwa7 | 1.22 | 1.88 | 1.28 | 0.68 | 0.85 | 0.47 |
| Adamts20 | 0.38 | 0.38 | 0.26 | 0.17 | 0.17 | 0.08 |
| Lgi2 | 7.48 | 1.17 | 1.13 | 10.83 | 8.87 | 9.33 |
| Fgf14 | 0.12 | 0.25 | 0.23 | 0.05 | 0.10 | 0.06 |
| Kera | 0.29 | 0.84 | 0.72 | 0.21 | 0.00 | 0.15 |
| Frzb | 0.25 | 1.02 | 0.63 | 0.18 | 0.24 | 0.26 |
| Grem1 | 1.37 | 1.76 | 1.97 | 2.36 | 3.88 | 4.66 |
| Rspo3 | 0.69 | 1.24 | 0.65 | 0.42 | 0.41 | 0.24 |
| Adm | 17.97 | 3.02 | 3.04 | 15.71 | 24.26 | 30.46 |
| Lif | 0.79 | 0.47 | 0.28 | 2.22 | 0.90 | 1.02 |
| Adamts4 | 2.37 | 1.85 | 0.83 | 4.00 | 3.05 | 3.68 |
| C1qtnf4 | 2.39 | 6.05 | 4.46 | 2.04 | 2.01 | 1.78 |
| Gfra4 | 1.76 | 1.48 | 1.79 | 2.41 | 4.55 | 4.44 |
| Ulk4 | 0.11 | 0.18 | 0.17 | 0.08 | 0.05 | 0.05 |
| Cap1 | 18.56 | 11.33 | 43.76 | 78.73 | 24.30 | 93.50 |
| Npnt | 5.11 | 7.65 | 6.29 | 0.65 | 4.64 | 2.92 |
| Apoc4 | 0.40 | 1.21 | 1.05 | 0.37 | 0.31 | 0.39 |
| Acan | 0.60 | 0.77 | 0.09 | 0.22 | 0.01 | 0.04 |
| Ccbe1 | 0.32 | 0.24 | 0.32 | 0.46 | 0.68 | 0.79 |
| Col12a1 | 6.27 | 2.51 | 1.89 | 10.33 | 8.18 | 4.42 |
| Apold1 | 1.69 | 0.70 | 0.35 | 2.48 | 2.11 | 2.49 |
| Tslp | 1.96 | 1.76 | 3.35 | 0.76 | 1.32 | 1.05 |
| Thsd4 | 0.21 | 0.23 | 0.25 | 0.16 | 0.08 | 0.07 |
| Ereg | 4.13 | 2.51 | 5.26 | 3.22 | 1.28 | 1.36 |
| Alb | 0.57 | 0.89 | 1.10 | 0.93 | 0.49 | 9.90 |
| Tfpi2 | 0.85 | 0.62 | 0.49 | 2.06 | 1.19 | 1.41 |
| Saa4 | 0.22 | 0.00 | 0.00 | 0.64 | 0.22 | 0.88 |
| Acrbp | 1.32 | 2.58 | 2.87 | 0.98 | 1.24 | 1.16 |
| Apln | 1.85 | 0.80 | 0.59 | 2.65 | 1.97 | 2.97 |
| Izumo4 | 1.02 | 1.45 | 2.74 | 0.53 | 0.83 | 0.99 |
| Sema3d | 0.32 | 0.53 | 0.61 | 0.29 | 0.23 | 0.20 |
| Scube2 | 0.33 | 0.44 | 0.56 | 0.22 | 0.22 | 0.16 |
| Sema3a | 0.16 | 0.17 | 0.18 | 0.25 | 0.43 | 0.40 |
| Wfdc18 | 2.02 | 1.21 | 0.78 | 4.31 | 2.97 | 2.11 |
| Bmp4 | 4.39 | 9.98 | 1.58 | 3.33 | 1.62 | 1.75 |
| Col4a3 | 0.18 | 0.29 | 0.24 | 0.16 | 0.07 | 0.10 |
| Comp | 0.38 | 0.43 | 0.42 | 0.14 | 0.22 | 0.22 |
| Shh | 2.13 | 3.27 | 0.68 | 1.08 | 0.02 | 0.03 |
| Timp1 | 142.34 | 19.71 | 23.81 | 112.23 | 135.32 | 224.99 |
| Apol9a | 0.25 | 0.41 | 0.35 | 0.14 | 0.22 | 0.13 |
| Gm128 | 0.70 | 0.99 | 1.25 | 0.62 | 0.52 | 0.25 |
| Scgb1b27 | 0.18 | 1.06 | 1.50 | 0.23 | 0.14 | 19.55 |
| Prrg4 | 0.66 | 0.14 | 0.36 | 1.19 | 0.89 | 0.77 |
| Pla2g2e | 4.87 | 7.65 | 6.71 | 5.46 | 1.58 | 2.53 |
| Wfikkn2 | 0.43 | 0.81 | 0.40 | 0.20 | 0.37 | 0.24 |
| Lgi3 | 0.24 | 0.39 | 0.35 | 0.22 | 0.17 | 0.09 |
| Nxph3 | 0.47 | 0.96 | 0.67 | 0.45 | 0.34 | 0.24 |
| Ccl7 | 49.11 | 9.00 | 9.67 | 47.36 | 30.76 | 74.16 |
| Gm13306 | 0.91 | 4.71 | 3.51 | 2.01 | 0.98 | 1.04 |
| Bdnf | 0.13 | 0.30 | 0.19 | 0.06 | 0.12 | 0.12 |
| Scgb2b27 | 0.06 | 0.44 | 1.01 | 0.07 | 0.07 | 10.34 |
| Ostn | 0.38 | 0.69 | 0.88 | 0.00 | 0.32 | 0.32 |

Supplemental table 6. The FPKM values of significantly differentiated extracellular space associated genes.

| gene_name | FPKM.IMQ5_LTH_1 | FPKM.IMQ5_LTH_2 | FPKM.IMQ5_LTH_3 | FPKM.IMQ5_H2O_1 | FPKM.IMQ5_H2O_2 | FPKM.IMQ5_H2O_3 |
| --- | --- | --- | --- | --- | --- | --- |
| Prl2c3 | 0.00 | 0.00 | 0.00 | 2.43 | 4.86 | 5.10 |
| Serpina3b | 20.82 | 104.32 | 76.08 | 0.97 | 0.39 | 0.32 |
| Serpina3j | 83.94 | 221.64 | 160.93 | 5.37 | 1.51 | 2.41 |
| 4930486L24Rik | 0.00 | 0.00 | 0.00 | 0.58 | 1.30 | 1.60 |
| Gm7298 | 0.91 | 2.68 | 1.83 | 0.00 | 0.00 | 0.03 |
| Rarres1 | 66.31 | 90.63 | 79.87 | 18.61 | 8.29 | 8.31 |
| Psapl1 | 155.80 | 198.60 | 208.46 | 48.39 | 31.48 | 32.99 |
| Saa1 | 6.94 | 2.36 | 2.41 | 33.13 | 34.44 | 41.16 |
| Ngp | 0.24 | 0.00 | 0.08 | 8.45 | 6.49 | 11.54 |
| Srgn | 22.81 | 10.07 | 9.85 | 78.98 | 122.41 | 161.41 |
| Spp1 | 5.52 | 2.58 | 2.59 | 17.51 | 59.47 | 75.22 |
| Cxcl2 | 7.19 | 0.87 | 1.51 | 67.28 | 150.76 | 143.49 |
| Cd14 | 23.42 | 12.89 | 8.44 | 74.06 | 168.98 | 184.19 |
| Gc | 0.00 | 0.00 | 0.00 | 0.27 | 0.48 | 1.38 |
| Wnt7b | 6.67 | 7.52 | 9.92 | 1.78 | 1.71 | 1.79 |
| Prl2c2 | 0.00 | 0.00 | 0.00 | 0.29 | 1.01 | 0.93 |
| Ccl3 | 9.90 | 1.47 | 2.45 | 65.03 | 94.33 | 142.81 |
| Thbs1 | 7.79 | 4.43 | 3.47 | 22.81 | 28.68 | 29.16 |
| Chit1 | 65.45 | 82.79 | 125.85 | 24.00 | 14.47 | 13.94 |
| Nos2 | 0.36 | 0.35 | 0.25 | 1.66 | 4.12 | 6.05 |
| Ces4a | 31.98 | 19.13 | 18.60 | 6.20 | 3.31 | 2.40 |
| Ccl4 | 6.04 | 0.96 | 1.85 | 35.74 | 57.43 | 138.26 |
| Sfrp4 | 7.70 | 4.92 | 8.02 | 1.17 | 1.17 | 1.70 |
| Pla2g7 | 14.76 | 11.04 | 10.66 | 38.89 | 42.28 | 56.09 |
| Retnlg | 10.36 | 0.58 | 0.94 | 203.19 | 216.88 | 347.50 |
| Saa2 | 6.81 | 1.97 | 2.63 | 21.83 | 29.72 | 29.01 |
| Il12a | 0.03 | 0.00 | 0.00 | 0.31 | 0.53 | 0.66 |
| Serpinb7 | 3.69 | 7.33 | 15.17 | 0.87 | 0.26 | 0.29 |
| Retnla | 24.93 | 30.77 | 66.76 | 2.49 | 3.39 | 6.98 |
| Serpine1 | 1.57 | 1.33 | 1.12 | 6.67 | 4.66 | 8.91 |
| Serpinb12 | 24.36 | 34.67 | 58.08 | 11.77 | 8.10 | 7.26 |
| Slurp2 | 5.69 | 26.11 | 24.20 | 1.59 | 0.42 | 0.36 |
| Gpt | 13.34 | 24.69 | 24.85 | 5.27 | 5.88 | 6.98 |
| Mmp8 | 1.94 | 0.13 | 0.37 | 12.99 | 18.53 | 30.78 |
| Orm1 | 61.13 | 74.74 | 95.08 | 29.66 | 21.70 | 23.96 |
| Selp | 3.04 | 2.09 | 1.63 | 12.37 | 8.05 | 7.51 |
| Cstdc4 | 2.29 | 0.15 | 0.32 | 15.57 | 23.67 | 36.55 |
| Slurp1 | 1.75 | 6.73 | 7.29 | 0.59 | 0.18 | 0.10 |
| Cp | 3.93 | 3.29 | 2.27 | 9.23 | 9.35 | 10.18 |
| Hmcn2 | 3.83 | 4.94 | 4.22 | 1.73 | 1.67 | 0.97 |
| C1qtnf3 | 3.46 | 8.06 | 3.61 | 1.00 | 0.80 | 0.59 |
| Cxcl13 | 91.98 | 112.30 | 62.04 | 232.36 | 316.88 | 524.67 |
| Serpina12 | 3.25 | 9.81 | 20.97 | 1.65 | 0.39 | 0.64 |
| Serpina3m | 4.68 | 2.53 | 2.21 | 11.13 | 17.21 | 15.52 |
| Ckm | 874.30 | 1173.72 | 1072.30 | 439.62 | 402.36 | 297.16 |
| Pglyrp1 | 3.31 | 1.05 | 1.48 | 9.40 | 11.82 | 14.09 |
| Defb14 | 43.59 | 18.60 | 33.69 | 165.67 | 133.87 | 122.93 |
| Tnf | 2.55 | 1.10 | 0.82 | 5.13 | 12.82 | 26.01 |
| Serpinb1c | 0.54 | 1.52 | 1.17 | 0.06 | 0.15 | 0.06 |
| Cilp | 24.99 | 25.76 | 26.77 | 11.30 | 11.41 | 8.61 |
| Lgr6 | 3.15 | 6.64 | 4.89 | 1.73 | 1.19 | 1.11 |
| Procr | 9.58 | 5.78 | 5.24 | 25.35 | 19.58 | 20.86 |
| Il1r1 | 7.78 | 6.28 | 5.01 | 23.48 | 17.19 | 15.40 |
| Slpi | 28.41 | 3.94 | 5.57 | 91.29 | 114.24 | 156.49 |
| Ppp1r1a | 17.45 | 30.24 | 28.16 | 6.25 | 9.74 | 6.17 |
| Ccl21b | 6.43 | 0.00 | 7.19 | 0.00 | 0.00 | 0.00 |
| Il4ra | 27.71 | 11.36 | 11.57 | 66.93 | 60.26 | 65.24 |
| Serpina3n | 307.57 | 171.40 | 179.66 | 580.64 | 725.62 | 799.91 |
| Fgf23 | 0.05 | 0.00 | 0.00 | 4.46 | 0.44 | 0.55 |
| Cxcl3 | 1.16 | 0.02 | 0.09 | 10.13 | 22.81 | 19.60 |
| Ighm | 41.85 | 53.44 | 43.84 | 12.06 | 15.40 | 23.44 |
| Gfra1 | 5.18 | 2.60 | 2.05 | 10.52 | 11.36 | 11.74 |
| Mmp9 | 5.05 | 0.85 | 2.26 | 10.83 | 19.35 | 25.03 |
| Angptl4 | 9.57 | 17.75 | 5.33 | 47.95 | 42.36 | 32.89 |
| Il1b | 30.04 | 2.31 | 3.73 | 98.52 | 228.51 | 260.10 |
| Lamc2 | 9.03 | 4.46 | 4.69 | 14.09 | 24.33 | 22.76 |
| Dkkl1 | 11.56 | 14.08 | 39.51 | 5.50 | 3.92 | 4.31 |
| Klk5 | 45.89 | 72.86 | 121.23 | 27.56 | 20.51 | 27.08 |
| Reg3g | 0.07 | 0.02 | 0.00 | 0.24 | 0.67 | 1.00 |
| Serpina3f | 0.21 | 0.58 | 0.10 | 2.95 | 2.11 | 2.25 |
| Cdcp3 | 0.17 | 0.26 | 0.33 | 0.06 | 0.03 | 0.03 |
| Myoc | 6.67 | 14.44 | 17.81 | 4.25 | 2.58 | 1.39 |
| Wnt3 | 3.53 | 4.21 | 6.59 | 1.48 | 0.71 | 1.17 |
| Tgfa | 0.42 | 1.97 | 2.20 | 9.28 | 7.94 | 6.96 |
| Chga | 0.79 | 2.05 | 3.65 | 0.30 | 0.17 | 0.36 |
| Clec3b | 83.02 | 144.33 | 134.91 | 51.01 | 44.90 | 52.34 |
| Ptprz1 | 0.70 | 0.39 | 0.51 | 1.83 | 2.09 | 1.44 |
| Reg1 | 0.30 | 0.05 | 0.00 | 1.45 | 2.63 | 2.63 |
| Osm | 1.61 | 0.27 | 0.30 | 3.47 | 8.79 | 9.34 |
| Csf3 | 0.77 | 0.00 | 0.17 | 10.45 | 6.02 | 10.90 |
| Il20 | 1.17 | 4.00 | 1.13 | 0.36 | 0.08 | 0.11 |
| Saa3 | 289.76 | 62.36 | 29.16 | 843.75 | 810.95 | 1468.77 |
| Ltf | 0.05 | 0.02 | 0.00 | 0.24 | 0.30 | 0.46 |
| H60c | 8.32 | 8.93 | 12.13 | 4.27 | 3.56 | 2.94 |
| Rtn4rl2 | 2.11 | 0.56 | 1.05 | 8.39 | 6.18 | 6.44 |
| F10 | 1.29 | 0.25 | 0.19 | 3.48 | 4.91 | 7.03 |
| Adamts15 | 3.98 | 3.28 | 1.94 | 6.36 | 8.80 | 9.81 |
| Ndp | 1.32 | 1.61 | 0.59 | 0.17 | 0.12 | 0.25 |
| Pon1 | 0.09 | 1.17 | 0.84 | 0.05 | 0.02 | 0.07 |
| Areg | 6.55 | 1.16 | 1.36 | 20.40 | 16.37 | 21.89 |
| Defb6 | 567.94 | 537.49 | 529.99 | 318.31 | 147.68 | 169.24 |
| Lrg1 | 152.25 | 55.93 | 67.87 | 267.64 | 307.37 | 314.57 |
| Ccl24 | 1.29 | 1.24 | 2.72 | 0.29 | 0.33 | 0.32 |
| Il1f5 | 25.80 | 34.76 | 52.66 | 18.12 | 10.85 | 13.53 |
| Hpx | 5.15 | 1.40 | 0.94 | 13.01 | 10.54 | 23.05 |
| Ramp1 | 6.21 | 11.86 | 8.32 | 3.72 | 3.12 | 2.77 |
| Anxa9 | 12.68 | 22.86 | 34.04 | 10.13 | 6.87 | 6.89 |
| Il1rap | 1.25 | 0.94 | 1.10 | 2.39 | 3.10 | 3.41 |
| Lrrn1 | 3.43 | 5.28 | 2.94 | 0.59 | 1.31 | 1.28 |
| Hp | 879.85 | 304.34 | 351.98 | 1743.16 | 1499.99 | 1760.64 |
| Clcf1 | 6.52 | 2.96 | 1.30 | 11.01 | 16.31 | 16.06 |
| Ptx3 | 3.49 | 0.72 | 0.79 | 9.39 | 6.94 | 11.27 |
| Acpp | 13.39 | 14.96 | 24.93 | 9.61 | 4.71 | 4.66 |
| Igfbp6 | 54.01 | 100.73 | 94.79 | 33.92 | 34.88 | 40.58 |
| Vwa2 | 4.20 | 7.50 | 5.05 | 2.20 | 2.17 | 2.09 |
| Mst1 | 3.70 | 6.19 | 6.53 | 1.69 | 1.78 | 1.67 |
| Serpinb3d | 0.23 | 0.27 | 0.06 | 1.11 | 2.34 | 0.83 |
| Nrg4 | 2.00 | 2.56 | 2.86 | 0.86 | 0.99 | 0.41 |
| Lrrc17 | 7.40 | 7.11 | 7.01 | 2.92 | 3.40 | 3.10 |
| Fgf7 | 2.41 | 1.83 | 1.35 | 5.62 | 5.38 | 5.48 |
| Nrg1 | 0.26 | 0.02 | 0.05 | 0.99 | 0.99 | 0.80 |
| Vcam1 | 0.81 | 0.70 | 0.59 | 1.59 | 2.51 | 2.03 |
| Cpa4 | 22.97 | 32.03 | 65.79 | 16.82 | 14.67 | 12.57 |
| Sema6b | 3.61 | 2.55 | 2.20 | 6.38 | 8.09 | 7.23 |
| Cxcl12 | 13.89 | 7.99 | 6.85 | 22.57 | 22.32 | 25.92 |
| Lcn2 | 94.10 | 9.78 | 11.72 | 311.05 | 239.95 | 248.74 |
| Hbegf | 5.42 | 1.86 | 1.79 | 13.37 | 8.11 | 8.57 |
| Lipg | 2.30 | 1.08 | 0.83 | 4.06 | 3.52 | 8.39 |
| Il18 | 15.03 | 28.56 | 45.96 | 10.50 | 10.74 | 9.52 |
| Ccl12 | 7.48 | 3.36 | 1.68 | 14.77 | 13.16 | 50.83 |
| Ccl6 | 194.76 | 50.98 | 85.05 | 266.66 | 425.37 | 451.92 |
| Ccl9 | 54.85 | 20.65 | 25.41 | 76.37 | 92.60 | 127.76 |
| Col4a6 | 0.39 | 0.86 | 0.68 | 0.24 | 0.11 | 0.07 |
| Scube3 | 2.30 | 1.04 | 0.98 | 0.59 | 0.23 | 0.31 |
| Il33 | 25.55 | 3.67 | 6.26 | 69.03 | 42.73 | 54.49 |
| Arg1 | 7.92 | 3.59 | 6.22 | 22.51 | 17.07 | 11.51 |
| Dmkn | 567.94 | 539.72 | 1018.24 | 337.59 | 298.32 | 320.94 |
| Cdsn | 100.30 | 119.96 | 237.60 | 63.76 | 62.80 | 64.50 |
| Mstn | 1.53 | 4.94 | 1.75 | 0.94 | 0.53 | 0.34 |
| Fgf6 | 0.30 | 0.27 | 0.25 | 0.05 | 0.09 | 0.01 |
| Ces2f | 1.60 | 0.89 | 1.61 | 4.37 | 4.01 | 3.42 |
| Ambp | 0.37 | 0.13 | 0.23 | 1.12 | 0.89 | 1.84 |
| Tnc | 58.25 | 9.45 | 11.44 | 104.48 | 123.91 | 122.07 |
| Rarres2 | 99.69 | 45.49 | 55.64 | 157.82 | 161.96 | 169.40 |
| Sele | 2.16 | 1.71 | 1.51 | 6.33 | 4.97 | 3.91 |
| Crispld2 | 9.90 | 5.47 | 6.72 | 13.44 | 17.31 | 19.29 |
| Cfb | 36.21 | 27.05 | 18.30 | 64.56 | 50.84 | 69.03 |
| Entpd1 | 9.31 | 6.11 | 5.76 | 13.71 | 21.18 | 12.91 |
| Sema4g | 2.39 | 4.70 | 3.89 | 2.09 | 1.29 | 1.05 |
| Tnfaip6 | 3.99 | 3.55 | 2.27 | 15.54 | 5.91 | 9.54 |
| Edn3 | 0.64 | 0.96 | 0.17 | 0.07 | 0.17 | 0.08 |
| Tril | 2.70 | 4.99 | 3.02 | 1.74 | 1.24 | 1.38 |
| Angptl1 | 2.09 | 2.63 | 2.36 | 0.93 | 0.74 | 0.85 |
| Enpp1 | 0.66 | 0.30 | 0.33 | 1.39 | 1.16 | 1.48 |
| S100a8 | 985.39 | 82.90 | 89.32 | 3174.95 | 2249.15 | 2502.52 |
| Wnt6 | 2.74 | 3.82 | 2.46 | 1.26 | 0.95 | 0.86 |
| Pamr1 | 1.14 | 3.06 | 3.67 | 0.43 | 0.84 | 1.05 |
| Wnt2 | 0.47 | 1.07 | 0.80 | 0.28 | 0.15 | 0.13 |
| Lgals3bp | 14.66 | 16.28 | 13.60 | 34.42 | 32.93 | 22.48 |
| Ptn | 5.32 | 12.21 | 6.19 | 4.45 | 2.20 | 1.75 |
| Klk14 | 10.56 | 2.11 | 2.29 | 29.82 | 17.93 | 15.83 |
| Anxa1 | 99.02 | 61.15 | 76.13 | 176.09 | 197.57 | 138.82 |
| Serpinb3b | 17.94 | 2.90 | 12.40 | 58.60 | 36.31 | 29.55 |
| Defb3 | 40.09 | 2.02 | 3.49 | 154.06 | 108.06 | 103.53 |
| Sema3e | 0.26 | 0.65 | 0.52 | 0.20 | 0.10 | 0.08 |
| Cxcl5 | 3.61 | 0.02 | 0.08 | 43.34 | 16.98 | 17.68 |
| Elfn1 | 0.17 | 0.04 | 0.06 | 0.43 | 0.43 | 0.46 |
| Wnt10b | 2.00 | 3.04 | 0.82 | 0.79 | 0.16 | 0.24 |
| Ctss | 93.05 | 81.51 | 72.44 | 136.47 | 157.06 | 216.31 |
| Prelp | 24.51 | 18.99 | 13.78 | 38.36 | 34.86 | 43.07 |
| Pcsk5 | 2.13 | 1.13 | 0.71 | 3.58 | 3.30 | 2.79 |
| Lpo | 7.83 | 3.58 | 4.55 | 2.98 | 1.31 | 1.11 |
| Man2a1 | 11.13 | 6.18 | 6.67 | 13.98 | 17.97 | 17.84 |
| Serpina1e | 0.00 | 0.22 | 0.14 | 0.92 | 0.26 | 4.56 |
| Csta2 | 4.00 | 0.96 | 1.73 | 7.80 | 11.20 | 7.14 |
| Il6 | 0.47 | 0.00 | 0.10 | 3.39 | 1.10 | 2.24 |
| Lrrc32 | 11.06 | 7.21 | 4.87 | 16.48 | 15.11 | 16.02 |
| Angpt2 | 1.86 | 1.87 | 1.40 | 3.70 | 4.12 | 4.23 |
| Olfm4 | 0.12 | 0.00 | 0.00 | 0.35 | 0.67 | 0.90 |
| Bmp2 | 1.39 | 3.15 | 2.59 | 1.10 | 0.62 | 0.85 |
| Thbd | 18.42 | 10.19 | 11.61 | 29.46 | 28.02 | 23.56 |
| Rbp4 | 9.91 | 14.93 | 11.78 | 5.70 | 2.96 | 6.68 |
| Defb1 | 56.06 | 22.79 | 36.32 | 101.45 | 85.46 | 67.73 |
| Ace2 | 9.87 | 9.36 | 12.91 | 7.54 | 5.05 | 2.72 |
| Col4a4 | 0.60 | 0.92 | 0.76 | 0.26 | 0.39 | 0.27 |
| Cxcl1 | 3.46 | 0.07 | 0.07 | 23.29 | 8.68 | 11.17 |
| S100a9 | 3262.79 | 205.23 | 275.72 | 8607.27 | 6276.23 | 6533.72 |
| Serpinb6d | 0.81 | 1.19 | 0.88 | 0.32 | 0.00 | 0.03 |
| Adamts5 | 4.69 | 2.40 | 2.44 | 6.13 | 6.10 | 7.02 |
| Tnfsf14 | 1.76 | 0.11 | 0.37 | 3.94 | 4.44 | 3.88 |
| Sfrp2 | 5.07 | 5.72 | 3.50 | 8.87 | 9.01 | 13.36 |
| Il1rl1 | 0.48 | 0.35 | 0.49 | 0.88 | 1.20 | 1.48 |
| Gkn3 | 0.90 | 2.40 | 1.62 | 0.11 | 0.52 | 0.55 |
| S100a7a | 3.55 | 0.44 | 0.15 | 10.75 | 7.81 | 7.33 |
| Emilin2 | 13.12 | 9.10 | 8.97 | 15.14 | 18.77 | 29.18 |
| Bmper | 0.97 | 0.34 | 0.59 | 2.62 | 1.67 | 1.46 |
| Serpinb1b | 0.18 | 0.00 | 0.05 | 0.64 | 0.46 | 0.39 |
| Scube1 | 0.55 | 0.71 | 0.38 | 0.29 | 0.15 | 0.05 |
| Defb4 | 7.46 | 0.31 | 0.49 | 23.16 | 23.65 | 12.60 |
| Ces1f | 2.26 | 4.05 | 2.52 | 1.20 | 1.17 | 1.42 |
| Raet1d | 0.32 | 0.17 | 0.17 | 0.45 | 0.96 | 1.00 |
| Apoc2 | 3.90 | 1.47 | 2.73 | 5.70 | 6.69 | 13.36 |
| Timp4 | 1.88 | 2.54 | 2.14 | 2.88 | 6.11 | 5.07 |
| Col27a1 | 1.25 | 1.65 | 0.74 | 0.73 | 0.48 | 0.44 |
| Gdnf | 0.36 | 0.51 | 0.35 | 0.76 | 1.22 | 1.37 |
| Spock1 | 0.23 | 0.43 | 0.17 | 0.09 | 0.10 | 0.10 |
| Agrp | 1.02 | 1.40 | 2.48 | 0.63 | 0.60 | 0.35 |
| Stc2 | 3.68 | 1.97 | 2.18 | 5.63 | 5.80 | 4.69 |
| Ngf | 2.40 | 2.02 | 2.33 | 5.65 | 4.54 | 6.11 |
| Serpina3i | 1.58 | 1.02 | 1.06 | 4.55 | 2.25 | 2.91 |
| H2-Q10 | 3.36 | 1.64 | 2.00 | 3.98 | 5.97 | 5.43 |
| Tfpi | 1.30 | 1.99 | 1.64 | 3.22 | 2.86 | 4.39 |
| Il23a | 0.25 | 0.12 | 0.00 | 0.92 | 0.58 | 0.72 |
| F5 | 0.18 | 0.11 | 0.04 | 0.26 | 0.45 | 0.37 |
| Mmp13 | 2.63 | 0.14 | 0.90 | 3.76 | 7.14 | 5.22 |
| Ppbp | 0.53 | 0.13 | 0.53 | 0.88 | 1.89 | 1.71 |
| Napsa | 4.56 | 1.82 | 2.03 | 4.47 | 6.56 | 10.10 |
| Col8a2 | 0.48 | 1.39 | 1.05 | 0.54 | 0.08 | 0.23 |
| Wnt3a | 1.54 | 1.16 | 1.35 | 2.74 | 3.31 | 3.23 |
| Svep1 | 5.54 | 2.18 | 2.00 | 5.52 | 6.99 | 7.96 |
| Cmtm8 | 1.87 | 2.74 | 1.77 | 1.28 | 0.63 | 0.91 |
| Lrp8 | 0.34 | 0.23 | 0.09 | 0.54 | 0.81 | 0.47 |
| Epgn | 9.89 | 0.20 | 1.34 | 28.29 | 17.52 | 16.72 |
| Pla1a | 4.68 | 2.49 | 3.39 | 4.35 | 7.23 | 15.92 |
| Glipr1 | 2.29 | 1.45 | 1.81 | 2.52 | 4.55 | 7.70 |
| Adamts20 | 0.38 | 0.38 | 0.26 | 0.17 | 0.17 | 0.08 |
| Lgi2 | 7.48 | 1.17 | 1.13 | 10.83 | 8.87 | 9.33 |
| Mmp25 | 0.35 | 0.09 | 0.10 | 0.52 | 0.59 | 0.58 |
| Frzb | 0.25 | 1.02 | 0.63 | 0.18 | 0.24 | 0.26 |
| Grem1 | 1.37 | 1.76 | 1.97 | 2.36 | 3.88 | 4.66 |
| Adm | 17.97 | 3.02 | 3.04 | 15.71 | 24.26 | 30.46 |
| Cpm | 5.65 | 5.99 | 9.07 | 6.23 | 1.79 | 1.89 |
| Lif | 0.79 | 0.47 | 0.28 | 2.22 | 0.90 | 1.02 |
| Adamts4 | 2.37 | 1.85 | 0.83 | 4.00 | 3.05 | 3.68 |
| C1qtnf4 | 2.39 | 6.05 | 4.46 | 2.04 | 2.01 | 1.78 |
| Gfra4 | 1.76 | 1.48 | 1.79 | 2.41 | 4.55 | 4.44 |
| Cap1 | 18.56 | 11.33 | 43.76 | 78.73 | 24.30 | 93.50 |
| Ggt1 | 0.40 | 0.47 | 0.08 | 2.02 | 0.52 | 0.68 |
| Npnt | 5.11 | 7.65 | 6.29 | 0.65 | 4.64 | 2.92 |
| Acan | 0.60 | 0.77 | 0.09 | 0.22 | 0.01 | 0.04 |
| Ccbe1 | 0.32 | 0.24 | 0.32 | 0.46 | 0.68 | 0.79 |
| Col12a1 | 6.27 | 2.51 | 1.89 | 10.33 | 8.18 | 4.42 |
| Tslp | 1.96 | 1.76 | 3.35 | 0.76 | 1.32 | 1.05 |
| Car2 | 1.78 | 0.95 | 1.58 | 2.97 | 4.41 | 2.41 |
| Serpinb3a | 33.93 | 8.49 | 11.20 | 70.92 | 36.27 | 21.98 |
| Ereg | 4.13 | 2.51 | 5.26 | 3.22 | 1.28 | 1.36 |
| Alb | 0.57 | 0.89 | 1.10 | 0.93 | 0.49 | 9.90 |
| Tfpi2 | 0.85 | 0.62 | 0.49 | 2.06 | 1.19 | 1.41 |
| Saa4 | 0.22 | 0.00 | 0.00 | 0.64 | 0.22 | 0.88 |
| Tnfrsf9 | 0.87 | 0.21 | 0.20 | 0.86 | 1.10 | 1.90 |
| Apln | 1.85 | 0.80 | 0.59 | 2.65 | 1.97 | 2.97 |
| Sema3d | 0.32 | 0.53 | 0.61 | 0.29 | 0.23 | 0.20 |
| Ltb | 2.17 | 0.96 | 0.40 | 2.16 | 3.07 | 4.26 |
| Scube2 | 0.33 | 0.44 | 0.56 | 0.22 | 0.22 | 0.16 |
| Sema3a | 0.16 | 0.17 | 0.18 | 0.25 | 0.43 | 0.40 |
| Serpina3k | 0.45 | 0.54 | 0.73 | 0.71 | 0.63 | 4.30 |
| Raet1e | 0.56 | 0.36 | 0.17 | 0.73 | 1.25 | 0.75 |
| Bmp4 | 4.39 | 9.98 | 1.58 | 3.33 | 1.62 | 1.75 |
| Col4a3 | 0.18 | 0.29 | 0.24 | 0.16 | 0.07 | 0.10 |
| Comp | 0.38 | 0.43 | 0.42 | 0.14 | 0.22 | 0.22 |
| Shh | 2.13 | 3.27 | 0.68 | 1.08 | 0.02 | 0.03 |
| Lingo3 | 0.41 | 0.70 | 0.90 | 0.20 | 0.37 | 0.37 |
| Cmtm5 | 0.26 | 1.01 | 1.23 | 0.38 | 0.32 | 0.29 |
| Timp1 | 142.34 | 19.71 | 23.81 | 112.23 | 135.32 | 224.99 |
| Adam10 | 10.70 | 2.08 | 6.71 | 13.54 | 11.96 | 13.68 |
| Hbb-bs | 912.23 | 466.76 | 855.88 | 150.99 | 777.70 | 138.08 |
| H2bc8 | 6.80 | 4.81 | 7.25 | 5.46 | 0.59 | 0.81 |
| Ctsw | 0.57 | 0.54 | 1.11 | 0.26 | 0.42 | 0.37 |
| Wfikkn2 | 0.43 | 0.81 | 0.40 | 0.20 | 0.37 | 0.24 |
| Hba-a2 | 701.46 | 390.60 | 761.22 | 119.45 | 663.57 | 117.09 |
| Ccl7 | 49.11 | 9.00 | 9.67 | 47.36 | 30.76 | 74.16 |
| Gm13306 | 0.91 | 4.71 | 3.51 | 2.01 | 0.98 | 1.04 |
| Hba-a1 | 734.75 | 393.80 | 799.63 | 122.02 | 700.65 | 120.67 |
| Bdnf | 0.13 | 0.30 | 0.19 | 0.06 | 0.12 | 0.12 |
| Scgb2b27 | 0.06 | 0.44 | 1.01 | 0.07 | 0.07 | 10.34 |
| Ostn | 0.38 | 0.69 | 0.88 | 0.00 | 0.32 | 0.32 |

Supplemental table 7. The FPKM values of significantly differentiated inflammatory system process associated genes.

| gene_name | FPKM.IMQ5_LTH_1 | FPKM.IMQ5_LTH_2 | FPKM.IMQ5_LTH_3 | FPKM.IMQ5_H2O_1 | FPKM.IMQ5_H2O_2 | FPKM.IMQ5_H2O_3 |
| --- | --- | --- | --- | --- | --- | --- |
| Gm49339 | 0.03 | 0.00 | 0.00 | 3.73 | 8.02 | 6.61 |
| Cd14 | 23.42 | 12.89 | 8.44 | 74.06 | 168.98 | 184.19 |
| Clec4d | 9.04 | 2.36 | 3.13 | 28.01 | 46.08 | 63.94 |
| Orm1 | 61.13 | 74.74 | 95.08 | 29.66 | 21.70 | 23.96 |
| Pla2g2f | 95.08 | 117.22 | 185.43 | 44.04 | 32.44 | 35.19 |
| Cd300lf | 1.35 | 0.17 | 0.21 | 6.06 | 10.95 | 11.03 |
| Ifitm1 | 59.72 | 19.31 | 19.21 | 143.06 | 175.32 | 255.61 |
| Pglyrp1 | 3.31 | 1.05 | 1.48 | 9.40 | 11.82 | 14.09 |
| Slpi | 28.41 | 3.94 | 5.57 | 91.29 | 114.24 | 156.49 |
| Lilrb4a | 6.95 | 3.37 | 3.96 | 11.33 | 19.37 | 23.20 |
| Il4ra | 27.71 | 11.36 | 11.57 | 66.93 | 60.26 | 65.24 |
| Ifi202b | 1.67 | 0.55 | 0.84 | 3.72 | 12.75 | 9.24 |
| Fgr | 5.90 | 2.26 | 1.72 | 11.63 | 14.59 | 22.97 |
| Treml4 | 0.25 | 0.09 | 0.22 | 0.90 | 1.87 | 3.69 |
| Nlrp3 | 1.11 | 0.30 | 0.31 | 1.49 | 11.46 | 8.22 |
| Gata3 | 13.71 | 17.92 | 14.56 | 8.27 | 4.95 | 4.61 |
| Cd300ld | 10.63 | 7.87 | 5.40 | 18.37 | 21.68 | 31.36 |
| Tlr13 | 3.00 | 1.97 | 1.63 | 5.54 | 6.83 | 9.48 |
| Ltf | 0.05 | 0.02 | 0.00 | 0.24 | 0.30 | 0.46 |
| H60c | 8.32 | 8.93 | 12.13 | 4.27 | 3.56 | 2.94 |
| Tarm1 | 1.47 | 0.27 | 0.46 | 4.17 | 4.64 | 6.87 |
| Mefv | 1.16 | 0.28 | 0.19 | 2.33 | 4.53 | 6.00 |
| Il1f5 | 25.80 | 34.76 | 52.66 | 18.12 | 10.85 | 13.53 |
| Il1rap | 1.25 | 0.94 | 1.10 | 2.39 | 3.10 | 3.41 |
| Mcoln2 | 2.54 | 0.99 | 1.21 | 5.94 | 5.36 | 6.47 |
| Hp | 879.85 | 304.34 | 351.98 | 1743.16 | 1499.99 | 1760.64 |
| Arid5a | 1.91 | 1.39 | 1.27 | 3.40 | 3.32 | 3.45 |
| Lcn2 | 94.10 | 9.78 | 11.72 | 311.05 | 239.95 | 248.74 |
| Clec4e | 1.17 | 0.12 | 0.20 | 1.47 | 3.77 | 10.85 |
| Olr1 | 0.12 | 0.05 | 0.05 | 0.32 | 0.39 | 0.48 |
| Fcgr1 | 7.52 | 8.35 | 4.54 | 15.53 | 11.89 | 29.96 |
| Arg1 | 7.92 | 3.59 | 6.22 | 22.51 | 17.07 | 11.51 |
| Clec7a | 3.94 | 0.97 | 1.49 | 4.93 | 10.49 | 12.32 |
| Cd177 | 0.91 | 0.02 | 0.07 | 2.36 | 6.49 | 6.88 |
| Cfb | 36.21 | 27.05 | 18.30 | 64.56 | 50.84 | 69.03 |
| Tril | 2.70 | 4.99 | 3.02 | 1.74 | 1.24 | 1.38 |
| S100a8 | 985.39 | 82.90 | 89.32 | 3174.95 | 2249.15 | 2502.52 |
| Clec5a | 0.72 | 0.40 | 0.42 | 1.40 | 1.67 | 2.17 |
| Traf3 | 0.80 | 0.41 | 0.45 | 1.27 | 1.56 | 2.70 |
| Ctps | 9.15 | 6.14 | 5.13 | 16.93 | 13.43 | 13.45 |
| Anxa1 | 99.02 | 61.15 | 76.13 | 176.09 | 197.57 | 138.82 |
| Cd244a | 0.84 | 0.28 | 0.28 | 1.24 | 1.55 | 3.14 |
| Pirb | 22.74 | 17.37 | 12.21 | 32.06 | 32.19 | 53.91 |
| Cd300a | 4.95 | 3.73 | 2.52 | 6.97 | 7.30 | 10.97 |
| Themis2 | 7.89 | 7.13 | 5.02 | 10.39 | 13.93 | 20.33 |
| Il31ra | 0.67 | 1.41 | 1.56 | 0.14 | 0.56 | 0.22 |
| Lyn | 12.99 | 9.51 | 5.24 | 17.76 | 16.20 | 35.00 |
| Tlr4 | 0.16 | 1.27 | 0.40 | 1.92 | 2.87 | 3.09 |
| Cd300c2 | 14.45 | 11.32 | 7.09 | 22.31 | 20.07 | 35.58 |
| Tlr6 | 1.41 | 0.76 | 0.80 | 2.08 | 2.67 | 4.07 |
| Tyrobp | 172.94 | 124.27 | 112.52 | 215.81 | 255.43 | 414.35 |
| Nod2 | 3.56 | 1.64 | 1.30 | 7.69 | 4.75 | 4.69 |
| Nlrp10 | 3.74 | 4.11 | 5.61 | 2.80 | 1.64 | 1.68 |
| Syk | 7.16 | 4.69 | 4.03 | 7.62 | 11.10 | 15.00 |
| S100a9 | 3262.79 | 205.23 | 275.72 | 8607.27 | 6276.23 | 6533.72 |
| Lyar | 5.45 | 4.63 | 5.22 | 13.67 | 8.82 | 10.08 |
| Cd300ld3 | 17.00 | 10.32 | 10.55 | 22.40 | 27.49 | 35.07 |
| Tlr2 | 6.56 | 3.54 | 3.25 | 6.11 | 10.55 | 17.19 |
| H2-DMb2 | 3.73 | 2.42 | 2.57 | 0.75 | 1.06 | 1.53 |
| Tlr1 | 4.92 | 3.91 | 1.86 | 6.21 | 6.90 | 13.09 |
| Ets1 | 2.44 | 1.61 | 2.43 | 3.25 | 5.05 | 4.74 |
| Tlr8 | 0.84 | 0.65 | 0.66 | 1.37 | 2.14 | 2.04 |
| H2-Q10 | 3.36 | 1.64 | 2.00 | 3.98 | 5.97 | 5.43 |
| Il23a | 0.25 | 0.12 | 0.00 | 0.92 | 0.58 | 0.72 |
| Cd7 | 4.20 | 2.99 | 3.04 | 1.10 | 1.08 | 1.90 |
| Krt16 | 2233.91 | 723.08 | 1326.59 | 3119.17 | 2877.52 | 3232.26 |
| Irf4 | 2.96 | 2.28 | 3.39 | 0.79 | 1.66 | 1.62 |
| Hck | 17.71 | 17.03 | 6.14 | 22.50 | 20.10 | 56.52 |
| Cd300lb | 3.06 | 1.93 | 1.71 | 4.06 | 4.70 | 6.17 |
| Jaml | 1.29 | 0.18 | 0.17 | 1.40 | 2.83 | 2.73 |
| Clec4n | 15.39 | 10.26 | 10.35 | 15.94 | 19.06 | 43.43 |
| Alcam | 2.81 | 4.64 | 2.91 | 2.76 | 1.05 | 0.97 |
| Tifa | 3.06 | 2.21 | 1.86 | 3.80 | 4.65 | 6.48 |
| Oas3 | 0.26 | 0.34 | 0.25 | 0.51 | 0.76 | 0.79 |
| Ggt1 | 0.40 | 0.47 | 0.08 | 2.02 | 0.52 | 0.68 |
| Tnfrsf17 | 0.86 | 1.47 | 1.28 | 0.26 | 0.72 | 0.39 |
| Padi4 | 1.39 | 0.64 | 0.86 | 2.62 | 1.76 | 1.98 |
| Naip2 | 1.39 | 0.90 | 0.83 | 1.44 | 2.44 | 2.60 |
| Slamf6 | 0.05 | 0.07 | 0.05 | 0.11 | 0.15 | 0.15 |
| Cd300e | 5.79 | 5.15 | 1.37 | 6.79 | 4.12 | 20.03 |
| March1 | 0.65 | 0.70 | 0.28 | 0.81 | 0.99 | 1.57 |
| Nlrc4 | 0.49 | 0.32 | 0.34 | 0.58 | 0.74 | 1.02 |

Supplemental table 8. The FPKM values of significantly differentiated chemokine signaling pathway associated genes.

| gene_name | FPKM.IMQ5_LTH_1 | FPKM.IMQ5_LTH_2 | FPKM.IMQ5_LTH_3 | FPKM.IMQ5_H2O_1 | FPKM.IMQ5_H2O_2 | FPKM.IMQ5_H2O_3 |
| --- | --- | --- | --- | --- | --- | --- |
| Cxcl2 | 7.19 | 0.87 | 1.51 | 67.28 | 150.76 | 143.49 |
| Ccl3 | 9.90 | 1.47 | 2.45 | 65.03 | 94.33 | 142.81 |
| Ccl4 | 6.04 | 0.96 | 1.85 | 35.74 | 57.43 | 138.26 |
| Cxcl13 | 91.98 | 112.30 | 62.04 | 232.36 | 316.88 | 524.67 |
| Cxcr2 | 4.28 | 1.09 | 1.37 | 9.72 | 18.02 | 16.03 |
| Ccr1 | 5.06 | 1.19 | 1.41 | 11.73 | 18.95 | 20.69 |
| Ccl21b | 6.43 | 0.00 | 7.19 | 0.00 | 0.00 | 0.00 |
| Fgr | 5.90 | 2.26 | 1.72 | 11.63 | 14.59 | 22.97 |
| Cxcl3 | 1.16 | 0.02 | 0.09 | 10.13 | 22.81 | 19.60 |
| Arrb1 | 0.77 | 2.24 | 0.66 | 2.48 | 10.80 | 9.88 |
| Nfkbia | 45.60 | 31.27 | 26.33 | 101.48 | 79.37 | 90.94 |
| Ccr5 | 2.10 | 1.67 | 0.91 | 5.04 | 3.68 | 5.59 |
| Ccl24 | 1.29 | 1.24 | 2.72 | 0.29 | 0.33 | 0.32 |
| Adcy1 | 0.36 | 1.01 | 1.00 | 0.25 | 0.16 | 0.17 |
| Cxcl12 | 13.89 | 7.99 | 6.85 | 22.57 | 22.32 | 25.92 |
| Ccl12 | 7.48 | 3.36 | 1.68 | 14.77 | 13.16 | 50.83 |
| Ccl6 | 194.76 | 50.98 | 85.05 | 266.66 | 425.37 | 451.92 |
| Ccl9 | 54.85 | 20.65 | 25.41 | 76.37 | 92.60 | 127.76 |
| Cxcl5 | 3.61 | 0.02 | 0.08 | 43.34 | 16.98 | 17.68 |
| Lyn | 12.99 | 9.51 | 5.24 | 17.76 | 16.20 | 35.00 |
| Cxcl1 | 3.46 | 0.07 | 0.07 | 23.29 | 8.68 | 11.17 |
| Crk | 4.24 | 10.12 | 9.22 | 17.17 | 16.42 | 15.59 |
| Cxcr1 | 0.30 | 0.00 | 0.00 | 0.66 | 1.00 | 1.73 |
| Hck | 17.71 | 17.03 | 6.14 | 22.50 | 20.10 | 56.52 |
| Was | 6.80 | 4.47 | 3.64 | 8.48 | 8.84 | 13.98 |
| Ppbp | 0.53 | 0.13 | 0.53 | 0.88 | 1.89 | 1.71 |
| Pik3r5 | 7.26 | 5.96 | 2.08 | 8.32 | 7.77 | 20.76 |
| Ccr4 | 0.31 | 0.26 | 0.59 | 0.16 | 0.06 | 0.00 |
| Vav1 | 8.70 | 7.32 | 4.11 | 10.54 | 8.82 | 23.69 |
| Cxcr4 | 5.01 | 5.92 | 2.62 | 5.91 | 10.58 | 11.95 |
| Ccl7 | 49.11 | 9.00 | 9.67 | 47.36 | 30.76 | 74.16 |
| Gm13306 | 0.91 | 4.71 | 3.51 | 2.01 | 0.98 | 1.04 |
| Ccr10 | 0.16 | 0.54 | 0.69 | 0.12 | 0.20 | 0.27 |

Supplemental table 9. The FPKM values of significantly differentiated cytokine-cytokine receptor interaction associated genes.

| gene_name | FPKM.IMQ5_LTH_1 | FPKM.IMQ5_LTH_2 | FPKM.IMQ5_LTH_3 | FPKM.IMQ5_H2O_1 | FPKM.IMQ5_H2O_2 | FPKM.IMQ5_H2O_3 |
| --- | --- | --- | --- | --- | --- | --- |
| Cxcl2 | 7.19 | 0.87 | 1.51 | 67.28 | 150.76 | 143.49 |
| Ccl3 | 9.90 | 1.47 | 2.45 | 65.03 | 94.33 | 142.81 |
| Ccl4 | 6.04 | 0.96 | 1.85 | 35.74 | 57.43 | 138.26 |
| Il12a | 0.03 | 0.00 | 0.00 | 0.31 | 0.53 | 0.66 |
| Il20ra | 2.22 | 2.64 | 3.87 | 0.50 | 0.18 | 0.30 |
| Cxcl13 | 91.98 | 112.30 | 62.04 | 232.36 | 316.88 | 524.67 |
| Acacb | 16.65 | 22.23 | 20.02 | 5.32 | 9.11 | 5.10 |
| Cxcr2 | 4.28 | 1.09 | 1.37 | 9.72 | 18.02 | 16.03 |
| Ccr1 | 5.06 | 1.19 | 1.41 | 11.73 | 18.95 | 20.69 |
| Tnf | 2.55 | 1.10 | 0.82 | 5.13 | 12.82 | 26.01 |
| Il1r1 | 7.78 | 6.28 | 5.01 | 23.48 | 17.19 | 15.40 |
| Ccl21b | 6.43 | 0.00 | 7.19 | 0.00 | 0.00 | 0.00 |
| Il4ra | 27.71 | 11.36 | 11.57 | 66.93 | 60.26 | 65.24 |
| Cxcl3 | 1.16 | 0.02 | 0.09 | 10.13 | 22.81 | 19.60 |
| Il1b | 30.04 | 2.31 | 3.73 | 98.52 | 228.51 | 260.10 |
| Irs1 | 3.52 | 4.62 | 3.80 | 1.28 | 1.71 | 1.65 |
| Socs3 | 32.29 | 15.00 | 13.18 | 62.72 | 65.64 | 72.11 |
| Nfkbia | 45.60 | 31.27 | 26.33 | 101.48 | 79.37 | 90.94 |
| Tnfrsf19 | 7.08 | 10.84 | 6.83 | 4.11 | 1.83 | 1.67 |
| Csf3 | 0.77 | 0.00 | 0.17 | 10.45 | 6.02 | 10.90 |
| Ccr5 | 2.10 | 1.67 | 0.91 | 5.04 | 3.68 | 5.59 |
| Il1r2 | 14.20 | 20.91 | 22.78 | 35.57 | 66.34 | 65.42 |
| Csf3r | 17.54 | 5.02 | 3.61 | 29.26 | 43.58 | 54.73 |
| Ccl24 | 1.29 | 1.24 | 2.72 | 0.29 | 0.33 | 0.32 |
| Il1f5 | 25.80 | 34.76 | 52.66 | 18.12 | 10.85 | 13.53 |
| Il1rap | 1.25 | 0.94 | 1.10 | 2.39 | 3.10 | 3.41 |
| Csf2rb2 | 7.34 | 6.52 | 4.96 | 12.67 | 16.18 | 13.58 |
| Clcf1 | 6.52 | 2.96 | 1.30 | 11.01 | 16.31 | 16.06 |
| Prlr | 0.39 | 0.66 | 0.57 | 0.12 | 0.12 | 0.20 |
| Cxcl12 | 13.89 | 7.99 | 6.85 | 22.57 | 22.32 | 25.92 |
| Ifngr1 | 8.79 | 6.32 | 5.76 | 12.22 | 16.59 | 21.60 |
| Il18 | 15.03 | 28.56 | 45.96 | 10.50 | 10.74 | 9.52 |
| Ccl12 | 7.48 | 3.36 | 1.68 | 14.77 | 13.16 | 50.83 |
| Ccl6 | 194.76 | 50.98 | 85.05 | 266.66 | 425.37 | 451.92 |
| Ccl9 | 54.85 | 20.65 | 25.41 | 76.37 | 92.60 | 127.76 |
| Il33 | 25.55 | 3.67 | 6.26 | 69.03 | 42.73 | 54.49 |
| Il17ra | 4.99 | 3.29 | 3.21 | 7.70 | 7.71 | 9.29 |
| Mstn | 1.53 | 4.94 | 1.75 | 0.94 | 0.53 | 0.34 |
| Acsbg1 | 34.70 | 70.08 | 66.32 | 27.02 | 27.65 | 26.01 |
| Edar | 0.73 | 1.59 | 1.03 | 0.29 | 0.01 | 0.14 |
| Il18rap | 1.22 | 0.09 | 0.26 | 2.16 | 4.13 | 3.47 |
| Cxcl5 | 3.61 | 0.02 | 0.08 | 43.34 | 16.98 | 17.68 |
| Il31ra | 0.67 | 1.41 | 1.56 | 0.14 | 0.56 | 0.22 |
| Il6 | 0.47 | 0.00 | 0.10 | 3.39 | 1.10 | 2.24 |
| Ppara | 0.59 | 0.48 | 0.42 | 0.19 | 0.17 | 0.10 |
| Bmp2 | 1.39 | 3.15 | 2.59 | 1.10 | 0.62 | 0.85 |
| Cxcl1 | 3.46 | 0.07 | 0.07 | 23.29 | 8.68 | 11.17 |
| Acvrl1 | 4.25 | 4.46 | 3.25 | 10.73 | 6.46 | 7.08 |
| Tnfsf14 | 1.76 | 0.11 | 0.37 | 3.94 | 4.44 | 3.88 |
| Il1rl1 | 0.48 | 0.35 | 0.49 | 0.88 | 1.20 | 1.48 |
| Eda2r | 2.26 | 1.53 | 2.26 | 0.79 | 0.96 | 0.99 |
| Il21r | 0.85 | 0.30 | 0.14 | 1.52 | 1.21 | 2.25 |
| Agrp | 1.02 | 1.40 | 2.48 | 0.63 | 0.60 | 0.35 |
| Ngf | 2.40 | 2.02 | 2.33 | 5.65 | 4.54 | 6.11 |
| Il23a | 0.25 | 0.12 | 0.00 | 0.92 | 0.58 | 0.72 |
| Cxcr1 | 0.30 | 0.00 | 0.00 | 0.66 | 1.00 | 1.73 |
| Ppbp | 0.53 | 0.13 | 0.53 | 0.88 | 1.89 | 1.71 |
| Prkag3 | 1.28 | 1.51 | 1.84 | 0.69 | 0.87 | 0.38 |
| Rxrg | 2.62 | 4.13 | 2.78 | 1.58 | 1.61 | 1.33 |
| Ccr4 | 0.31 | 0.26 | 0.59 | 0.16 | 0.06 | 0.00 |
| Lif | 0.79 | 0.47 | 0.28 | 2.22 | 0.90 | 1.02 |
| Ackr4 | 0.60 | 2.36 | 2.28 | 0.96 | 0.61 | 0.64 |
| Cxcr4 | 5.01 | 5.92 | 2.62 | 5.91 | 10.58 | 11.95 |
| Tnfrsf17 | 0.86 | 1.47 | 1.28 | 0.26 | 0.72 | 0.39 |
| Tslp | 1.96 | 1.76 | 3.35 | 0.76 | 1.32 | 1.05 |
| Tnfrsf9 | 0.87 | 0.21 | 0.20 | 0.86 | 1.10 | 1.90 |
| Il24 | 3.59 | 0.00 | 0.20 | 9.99 | 8.34 | 10.16 |
| Ltb | 2.17 | 0.96 | 0.40 | 2.16 | 3.07 | 4.26 |
| Amhr2 | 1.66 | 1.71 | 1.35 | 0.41 | 1.16 | 0.58 |
| Bmp4 | 4.39 | 9.98 | 1.58 | 3.33 | 1.62 | 1.75 |
| Il11ra2 | 2.54 | 1.90 | 0.90 | 4.43 | 3.10 | 3.67 |
| Gm13305 | 0.61 | 0.70 | 0.98 | 1.92 | 1.17 | 1.50 |
| Ccl7 | 49.11 | 9.00 | 9.67 | 47.36 | 30.76 | 74.16 |
| Gm13306 | 0.91 | 4.71 | 3.51 | 2.01 | 0.98 | 1.04 |
| Ccr10 | 0.16 | 0.54 | 0.69 | 0.12 | 0.20 | 0.27 |

Supplemental table 10. The FPKM values of significantly differentiated IL-17 signaling pathway associated genes.

| gene_name | FPKM.IMQ5_LTH_1 | FPKM.IMQ5_LTH_2 | FPKM.IMQ5_LTH_3 | FPKM.IMQ5_H2O_1 | FPKM.IMQ5_H2O_2 | FPKM.IMQ5_H2O_3 |
| --- | --- | --- | --- | --- | --- | --- |
| Cxcl2 | 7.19 | 0.87 | 1.51 | 67.28 | 150.76 | 143.49 |
| Tnf | 2.55 | 1.10 | 0.82 | 5.13 | 12.82 | 26.01 |
| Ptgs2 | 0.97 | 0.09 | 0.15 | 4.87 | 6.91 | 6.14 |
| Cxcl3 | 1.16 | 0.02 | 0.09 | 10.13 | 22.81 | 19.60 |
| Mmp9 | 5.05 | 0.85 | 2.26 | 10.83 | 19.35 | 25.03 |
| Il1b | 30.04 | 2.31 | 3.73 | 98.52 | 228.51 | 260.10 |
| Nfkbia | 45.60 | 31.27 | 26.33 | 101.48 | 79.37 | 90.94 |
| Csf3 | 0.77 | 0.00 | 0.17 | 10.45 | 6.02 | 10.90 |
| Fosl1 | 0.71 | 0.08 | 0.15 | 3.65 | 2.23 | 2.81 |
| Mapk13 | 37.27 | 56.76 | 65.02 | 25.76 | 23.45 | 21.18 |
| Lcn2 | 94.10 | 9.78 | 11.72 | 311.05 | 239.95 | 248.74 |
| Ccl12 | 7.48 | 3.36 | 1.68 | 14.77 | 13.16 | 50.83 |
| Il17ra | 4.99 | 3.29 | 3.21 | 7.70 | 7.71 | 9.29 |
| S100a8 | 985.39 | 82.90 | 89.32 | 3174.95 | 2249.15 | 2502.52 |
| Traf3 | 0.80 | 0.41 | 0.45 | 1.27 | 1.56 | 2.70 |
| Mmp3 | 73.22 | 43.34 | 33.72 | 79.39 | 127.09 | 171.61 |
| Cxcl5 | 3.61 | 0.02 | 0.08 | 43.34 | 16.98 | 17.68 |
| Il6 | 0.47 | 0.00 | 0.10 | 3.39 | 1.10 | 2.24 |
| Cxcl1 | 3.46 | 0.07 | 0.07 | 23.29 | 8.68 | 11.17 |
| S100a9 | 3262.79 | 205.23 | 275.72 | 8607.27 | 6276.23 | 6533.72 |
| Defb4 | 7.46 | 0.31 | 0.49 | 23.16 | 23.65 | 12.60 |
| Mmp13 | 2.63 | 0.14 | 0.90 | 3.76 | 7.14 | 5.22 |
| Mapk15 | 0.79 | 1.01 | 0.73 | 0.32 | 0.61 | 0.17 |
| Ccl7 | 49.11 | 9.00 | 9.67 | 47.36 | 30.76 | 74.16 |

Supplemental table 11. The FPKM values of significantly differentiated NF-kappa B signaling pathway associated genes.

| gene_name | FPKM.IMQ5_LTH_1 | FPKM.IMQ5_LTH_2 | FPKM.IMQ5_LTH_3 | FPKM.IMQ5_H2O_1 | FPKM.IMQ5_H2O_2 | FPKM.IMQ5_H2O_3 |
| --- | --- | --- | --- | --- | --- | --- |
| Cxcl2 | 7.19 | 0.87 | 1.51 | 67.28 | 150.76 | 143.49 |
| Cd14 | 23.42 | 12.89 | 8.44 | 74.06 | 168.98 | 184.19 |
| Ccl4 | 6.04 | 0.96 | 1.85 | 35.74 | 57.43 | 138.26 |
| Tnf | 2.55 | 1.10 | 0.82 | 5.13 | 12.82 | 26.01 |
| Ptgs2 | 0.97 | 0.09 | 0.15 | 4.87 | 6.91 | 6.14 |
| Il1r1 | 7.78 | 6.28 | 5.01 | 23.48 | 17.19 | 15.40 |
| Ccl21b | 6.43 | 0.00 | 7.19 | 0.00 | 0.00 | 0.00 |
| Il1b | 30.04 | 2.31 | 3.73 | 98.52 | 228.51 | 260.10 |
| Nfkbia | 45.60 | 31.27 | 26.33 | 101.48 | 79.37 | 90.94 |
| Vcam1 | 0.81 | 0.70 | 0.59 | 1.59 | 2.51 | 2.03 |
| Cxcl12 | 13.89 | 7.99 | 6.85 | 22.57 | 22.32 | 25.92 |
| Traf3 | 0.80 | 0.41 | 0.45 | 1.27 | 1.56 | 2.70 |
| Lyn | 12.99 | 9.51 | 5.24 | 17.76 | 16.20 | 35.00 |
| Syk | 7.16 | 4.69 | 4.03 | 7.62 | 11.10 | 15.00 |
| Tnfsf14 | 1.76 | 0.11 | 0.37 | 3.94 | 4.44 | 3.88 |
| Bcl2a1b | 5.07 | 3.78 | 2.06 | 7.32 | 8.04 | 20.69 |
| Malt1 | 0.16 | 0.08 | 0.13 | 0.97 | 0.24 | 0.20 |
| Tirap | 1.93 | 1.35 | 1.19 | 2.96 | 3.35 | 3.12 |
| Ltb | 2.17 | 0.96 | 0.40 | 2.16 | 3.07 | 4.26 |

Supplemental table 12. The FPKM values of significantly differentiated TNF-A signaling pathway associated genes.

| gene_name | FPKM.IMQ5_LTH_1 | FPKM.IMQ5_LTH_2 | FPKM.IMQ5_LTH_3 | FPKM.IMQ5_H2O_1 | FPKM.IMQ5_H2O_2 | FPKM.IMQ5_H2O_3 |
| --- | --- | --- | --- | --- | --- | --- |
| Cxcl2 | 7.19 | 0.87 | 1.51 | 67.28 | 150.76 | 143.49 |
| Tnf | 2.55 | 1.10 | 0.82 | 5.13 | 12.82 | 26.01 |
| Ptgs2 | 0.97 | 0.09 | 0.15 | 4.87 | 6.91 | 6.14 |
| Cxcl3 | 1.16 | 0.02 | 0.09 | 10.13 | 22.81 | 19.60 |
| Mmp9 | 5.05 | 0.85 | 2.26 | 10.83 | 19.35 | 25.03 |
| Il1b | 30.04 | 2.31 | 3.73 | 98.52 | 228.51 | 260.10 |
| Socs3 | 32.29 | 15.00 | 13.18 | 62.72 | 65.64 | 72.11 |
| Nfkbia | 45.60 | 31.27 | 26.33 | 101.48 | 79.37 | 90.94 |
| Mapk13 | 37.27 | 56.76 | 65.02 | 25.76 | 23.45 | 21.18 |
| Vcam1 | 0.81 | 0.70 | 0.59 | 1.59 | 2.51 | 2.03 |
| Ccl12 | 7.48 | 3.36 | 1.68 | 14.77 | 13.16 | 50.83 |
| Sele | 2.16 | 1.71 | 1.51 | 6.33 | 4.97 | 3.91 |
| Junb | 101.97 | 51.32 | 55.34 | 158.25 | 175.60 | 155.46 |
| Bcl3 | 18.75 | 10.79 | 8.56 | 31.45 | 25.34 | 29.72 |
| Traf3 | 0.80 | 0.41 | 0.45 | 1.27 | 1.56 | 2.70 |
| Mmp3 | 73.22 | 43.34 | 33.72 | 79.39 | 127.09 | 171.61 |
| Il6 | 0.47 | 0.00 | 0.10 | 3.39 | 1.10 | 2.24 |
| Nod2 | 3.56 | 1.64 | 1.30 | 7.69 | 4.75 | 4.69 |
| Cxcl1 | 3.46 | 0.07 | 0.07 | 23.29 | 8.68 | 11.17 |
| Mlkl | 4.37 | 3.08 | 3.55 | 6.92 | 7.05 | 8.18 |
| Lif | 0.79 | 0.47 | 0.28 | 2.22 | 0.90 | 1.02 |

Supplemental table 12. The data of KEGG enrichment analysis in metabolic pathway.

| KEGG_ID | Pathway_Name | S_Gene_Number | TS_Gene_Number | B_Gene_Number | TB_Gene_Number | p_Value | RichFactor |
| --- | --- | --- | --- | --- | --- | --- | --- |
| mmu00640 | Propanoate metabolism | 3 | 26 | 36 | 6328 | 0.00040191 | 0.08333333 |
| mmu00360 | Phenylalanine metabolism | 3 | 26 | 46 | 6328 | 0.00083151 | 0.06521739 |
| mmu00230 | Purine metabolism | 2 | 26 | 92 | 6328 | 0.05422251 | 0.02173913 |
| mmu00190 | Oxidative phosphorylation | 1 | 26 | 16 | 6328 | 0.06382547 | 0.0625 |
| mmu00020 | Citrate cycle (TCA cycle) | 1 | 26 | 20 | 6328 | 0.07915898 | 0.05 |
| mmu00232 | Caffeine metabolism | 1 | 26 | 21 | 6328 | 0.08295446 | 0.04761905 |
| mmu00250 | Alanine, aspartate and glutamate metabolism | 1 | 26 | 24 | 6328 | 0.09425085 | 0.04166667 |
| mmu00650 | Butanoate metabolism | 1 | 26 | 40 | 6328 | 0.15227429 | 0.025 |
| mmu00280 | Valine, leucine and isoleucine degradation | 1 | 26 | 41 | 6328 | 0.15577952 | 0.02439024 |
| mmu00630 | Glyoxylate and dicarboxylate metabolism | 1 | 26 | 44 | 6328 | 0.16621178 | 0.02272727 |
| mmu00760 | Nicotinate and nicotinamide metabolism | 1 | 26 | 44 | 6328 | 0.16621178 | 0.02272727 |
| mmu00240 | Pyrimidine metabolism | 1 | 26 | 59 | 6328 | 0.21654096 | 0.01694915 |
| mmu00350 | Tyrosine metabolism | 1 | 26 | 76 | 6328 | 0.27005003 | 0.01315789 |
| mmu01100 | Metabolic pathways | 5 | 26 | 1455 | 6328 | 0.74733437 | 0.00343643 |
| mmu01110 | Biosynthesis of secondary metabolites | 3 | 26 | 1023 | 6328 | 0.81583428 | 0.00293255 |
