# Supplementary material for: L-Theanine Alleviates IMQ-Induced Psoriasis Like Skin Inflammation by Downregulating the Production of IL-23 and Chemokines
Source: Front Pharmacol. 2021 Jul 26;12:719842. doi: 10.3389/fphar.2021.719842 (PMC8350042; doi:10.3389/fphar.2021.719842)
Supplement: Supplementary file 2 [file DataSheet1.docx]

**Supplementary data**

Supplementary figure 1. L-THE regulate the expression of cytokine-cytokine receptor interaction, chemokine TNF, NF-κB and IL-17A signaling pathway associated genes.


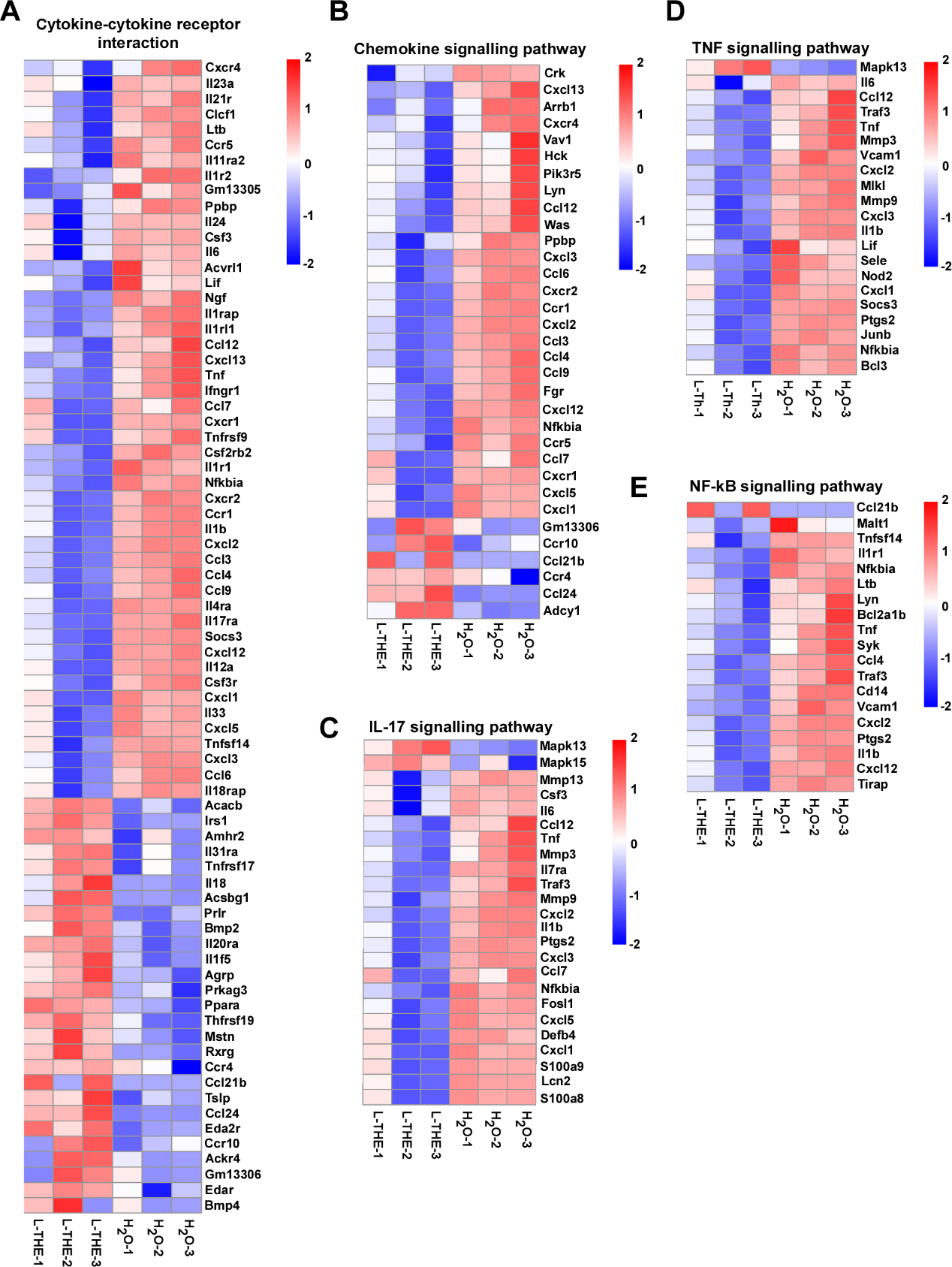


(A-E)The heatmap showed expression of cytokine-cytokine receptor interaction (A), chemokine (B), IL-17A (C), TNF (D), and NF-κB (E) signaling pathway associated genes based on RNA-seq data from IMQ-induced psoriasis mice treated with 100 mM L-THE or H_2_O for 5 days.
